# Identification of Differentially Expressed Genes Associated with the Prognosis and Diagnosis of Hepatocellular Carcinoma by Integrated Bioinformatics Analysis

**DOI:** 10.1155/2022/4237633

**Published:** 2022-10-22

**Authors:** Mohib Ullah Kakar, Muhammad Zubair Mehboob, Muhammad Akram, Muddaser Shah, Yasmeen Shakir, Hafza Wajeeha Ijaz, Ubair Aziz, Zahid Ullah, Sajjad Ahmad, Sikandar Ali, Yongxiang Yin

**Affiliations:** ^1^Beijing Key Laboratory for Separation and Analysis in Biomedicine and Pharmaceutical, School of life Sciences, Beijing Institute of Technology (BIT), Beijing 100081, China; ^2^Faculty of Marine Sciences, Lasbela University of Agriculture, Water and Marine Sciences (LUAWMS), Uthal, Balochistan, Pakistan; ^3^CAS Centre for Excellence in Biotic Interaction, College of Life Sciences, University of Chinese Academy of Science, Beijing 100049, China; ^4^Department of Biochemistry and Biotechnology, University of Gujrat, Gujrat 50700, Pakistan; ^5^School of Science, Department of Life sciences, University of Management and Technology, Johar Town, Lahore 54770, Pakistan; ^6^Department of Botany, Abdul Wali Khan University, Mardan 23200, Pakistan; ^7^Natural and Medical Sciences Research Center, University of Nizwa, Birkat Al-Mauz, P.O. Box 33, Nizwa 616, Oman; ^8^Department of Biochemistry, Hazara University, Mansehra, Pakistan; ^9^Research Centre of Molecular Simulation, National University of Science and Technology, Islamabad, Pakistan; ^10^School of Environmental Studies, China University of Geosciences, Wuhan 430074, China; ^11^Faculty of Veterinary and Animal Sciences, Lasbela University of Agriculture, Water and Marine Sciences, LUAWMS, Uthal, 90150 Balochistan, Pakistan; ^12^Dow Institute for Advanced Biological and Animal Research, Dow University of Health Sciences, Ojha Campus, Karachi, Pakistan; ^13^Department of Pathology, Wuxi Maternity and Child Health Hospital Affiliated to Nanjing Medical University, Wuxi, China

## Abstract

**Objective:**

The goal of this study was to understand the possible core genes associated with hepatocellular carcinoma (HCC) pathogenesis and prognosis.

**Methods:**

GEO contains datasets of gene expression, miRNA, and methylation patterns of diseased and healthy/control patients. The GSE62232 dataset was selected by employing the server Gene Expression Omnibus. A total of 91 samples were collected, including 81 HCC and 10 healthy samples as control. GSE62232 was analysed through GEO2R, and Functional Enrichment Analysis was performed to extract rational information from a set of DEGs. The Protein-Protein Relationship Networking search method has been used for extracting the interacting genes. MCC method was used to calculate the top 10 genes according to their importance. Hub genes in the network were analysed using GEPIA to estimate the effect of their differential expression on cancer progression.

**Results:**

We identified the top 10 hub genes through CytoHubba plugin. These included BUB1, BUB1B, CCNB1, CCNA2, CCNB2, CDC20, CDK1 and MAD2L1, NCAPG, and NDC80. NCAPG and NDC80 reported for the first time in this study while the remaining from a recently reported literature. The pathogenesis of HCC may be directly linked with the aforementioned genes. In this analysis, we found critical genes for HCC that showed recommendations for future prognostic and predictive biomarkers studies that could promote selective molecular therapy for HCC.

## 1. Introduction

Cancer, also known as malignancy, is characterized by the irregular cellular growth. More than 100 different types of cancers exist, in which most common are breast, skin, prostate, lungs, colon, and lymphoma [1]. Cancer is present in human as the most considerable public health concern worldwide, and liver cancer adds greatly to the morbidity and mortality in cancer [2]. Liver cancer (hepatocellular carcinoma) is the fourth leading cause of cancer-related death globally, ranking sixth in prevalence [3, 4]. Hepatocellular carcinoma (HCC) constitutes about 85–90% of all primary malignant liver tumors. Chronic hepatitis B virus (HBV) infection, hepatitis C virus (HCV), smoking, aflatoxin, obesity, chronic liver disease, and type 2 diabetes are the main risk factors [3, 5–7]. Of these variables, recurrent liver disease is the primary cause of liver cancer [8].

The prevalence of viral infection in HCC cases varies from developed to developing nations, where HBV reflects 60% in developing nations and 23% in developed nations, while HCV infection is responsible for 23% in emerging nations and 20% of patients in developed nations [9]. Moreover, the highest incidence of HBV is in sub-Saharan Africa, South-eastern Asia, and East Asia, while HCV is high in the USA, Europe, and Japan [8]. The prevalence of nonalcoholic fatty liver disease (NAFLD) also adversely affects individual health, causing increased obesity and other metabolic disorders [10]. Around 25-30% of patients having a western lifestyle possess more fats in their liver, 2-5% of which have NAFLD, and 1-2% suffer from non-alcoholic steatohepatitis cirrhosis [11].

The World Health Organization (WHO) estimates that in 2030 over one million people are going to die of liver cancer [12]. The key factor that affects HCC mortality is the poor diagnosis, which results in just 18% survival rate [13] less than the cancers of the breast (77.1%), renal pelvic (74.8%), and myeloma (52.2%). The high risk of recurrence and metastasis of the HCCs also contribute to a shorter life span and poor survival after hepatectomy [14]. Different variables participate in the HCC diagnoses, such as cell proliferation, apoptosis, and genes linked to the mTOR pathway. Different variables participate [15]. HCC is on a global increase, but early detection and therapy of HCC remain a concern [8]. In developing countries, the HCC prevalence is growing as a consequence of low levels of health and treatment, with a global rate of liver cancer per 100000 people approximately at 9.3 in 2018 [16], as well as poor prognosis [17].

The diverse factors implicated in liver cancer are cellular tumor antigen p53 (TP53), axin-1 (AXIN1), catenin *β*-1 (CTNNB1), and telomerase reverse transcriptase (TERT) promoters as well as other primary genes for mutation generation, p53 cell cycle system, WNT/*β*-catenin, oxidative stress, RAS/RAF/MAPK, and PI3K/AKT/MTOR pathways along with other main primary signaling pathways. Liu et al. used highly efficient microarray technology to screen molecular indicators across all human cancerous tumors, especially for liver cancer, by using Gene Expression Omnibus (GEO) datasets and The Cancer Genome Atlas (TCGA) RNA-sequence, and analysed with the help of bioinformatics methods [18–22]. Gene chip technology can also reliably represent the molecular expression profile and detect genetic variants correlated with HCC in liver cancer studies [23, 24]. The data, information, knowledge, and wisdom (DIKW) model is widely used in life science, including medicine [25–27]. Recently, genome-wide screening has significantly improved the knowledge of the genetic context and pathways that lead to the HCC [28–31].

Four core genes and two essential pathways of developing HCC from cirrhosis have been established by GEO dataset using a bioinformatics methodology, including DEG screening and networking of protein-protein interactions (PPIs) [32]. Zhang et al. screened the genes and pathways associated with HCC development and prevalence through a series of bioinformatics observations, such as DEG recognition, functional enrichment analysis, PPI network and module analysis, and weighted network correlation analysis [24]. Zhou et al. identified HCC critical genes and microRNAs through raw data processing by using Gene Ontology (GO), GEO2R, and Kyoto Encyclopaedia of Genes and Genomes (KEGG) pathway enrichment processing and PPI network creation [33, 34]. Li et al. identified 89 out of 320 consistent differentially expressed genes in HCC patients. The five most expressed genes include Collagen alpha-2(I) chain (*COL1A2*), osteopontin (*SPP1*), lipoprotein A (*LPA*), Insulin-like growth factor I (*IGF1*), and Galectin-3 (*LGALS3*) [35]. Another study characterized the 247 upregulated and 516 downregulated DEGs which were predominantly enhanced in the oxidation-reduction process, epoxygenase P450 pathway, and metabolism-related pathways. Investigations have shown that CDC20, CDK1, MAD2L1, BUB1, BUB1B, CCNB1, and CCNA2 are linked to the poor overall survival of HCC patients [36]. Meng et al. identified 11 hub genes as closely connected to pathogenesis and HCC prognosis (CCNB2, CDK1, CCNB1, CDC20, CCNA2, TOP2A, MELK, TPX2, PBK, KIF20A, and AURKA) [37]. Yan and Liu identified five hub genes CCNA2, PLK1, CDC20, UBE2C, and AURKA of hepatic cancer, which were dramatically elevated in the Cancer Genome Atlas [19]. Zhang et al. screened 293 frequent DEGs, comprising 103 upregulated and 190 downregulated genes. CDK1, TPX2, AURKA, CCNA2, KIF11, HMMR, BUB1B, TOP2A, TPX2, and CDC45 were the top 10 hub genes found in HCC of Chinese population [20]. The methylation role in gene expression was identified from 162 hypermethylated genes (downregulated) and 190 hypomethylated genes (upregulated). In biological processes, such as keratinocyte growth and calcium homeostasis, overregulated genes with poor methylation were identified [38]. PTK2, ITGA2, and VWF were found as highly expressing hypomethylated hub genes detected in the PPI network [38]. Three gene methylation levels, KPNA2, MCM3, and LRRC1, were linked to HCC clinical characteristics [39].

Applied bioinformatics research with the current genomic evidence offers an in-depth insight into therapeutic resistance and disease progression processes. This study focuses on the expression profiling of HCC patients compared to healthy ones. The GSE62232 dataset (GEO: https://www.ncbi.nlm.nih.gov/geo/) has been chosen. GSE62232 was analysed through GEO2R (https://www.ncbi.nlm.nih.gov/geo/geo2R) [40] to evaluate and recalculate the genes that are differentially expressed in healthy and diseased samples. However, new prognostic biomarkers are needed to improve HCC diagnosis and treatment.

## 2. Results

### 2.1. DEG Identification

**Figure 1:**
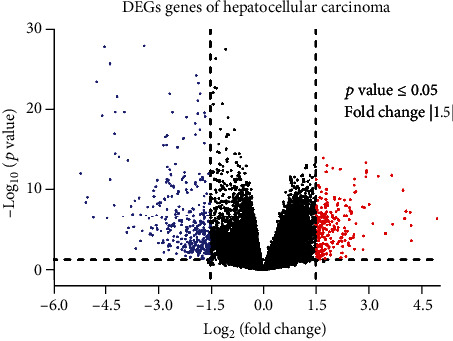
Volcano plot of the DEGs in HCC patients compared with control from the GSE62232 dataset. Each black dot represents one gene. The black and colorful dots above and side of the dotted line corresponds to those genes having *p* value ≤ 0.05 and fold change |1.5|. Blue dots are downregulated, and red dots are upregulated genes.

Expression profile of genes for GSE62232, titled in NCBI as “Large-Scale Gene Expression Profiling of 81 Hepatocellular Carcinomas” was obtained from the GEO dataset, which was generated on the GPL570 platform (Affymetrix Human Genome U133 Plus 2.0 Array). After getting data of 91 liver samples (81 HCC patients and 10 control) of GSE62232 study, the data was analysed through GEO2R to identify the DEGs of both upregulated and downregulated genes using a *p* value of ≤0.05 and log FC > 1.5 as selection criteria. Overall, 598 genes out of a total 19982 were differentially expressed in HCC samples with 233 upregulated and 365 downregulated genes (Supplementary Table 1). The DEGs are represented by a volcano plot constructed using Prism (http://www.graphpad.com/scientific-software/prism) for a better graphical representation of overall genes (Figure 1).

### 2.2. Enrichment Analysis of DEGs

In order to explore GO terms and cellular mechanisms affected by these DEGs, both over- and downregulated genes were imported into an online DAVID server to conduct the annotation process. The annotated GO terms were divided into MF, BP, and CC ontologies (*p* value < 0.05, FDR < 0.05). The GO BP analysis revealed that the majority of DEGs were enriched into oxidation-reduction (GO:0055114), metabolic (steroids, drug, and xenobiotic) and catabolic (exogenous drug, tryptophan), cell division (GO:0051301), response to the drug (GO:0042493), and p450 pathways (GO:0097267). For the GO MF analysis, most of the genes were significantly enriched in oxygen (GO:0019825) and iron (GO:0005506) ion binding, oxidoreductase activity (GO:0016705), monooxygenase activity (GO:0004497), heme binding (GO:0020037), steroid hydroxylase (GO:0008395), and electron carrier activity (GO:0009055). Concerning the GO CC analysis, commonly DEGs were enriched in extracellular space (GO:0070062, GO:0005615), organelle membrane (GO:0031090), and condensed chromosome kinetochore (GO:0000777) (Table 1).

The KEGG signaling pathway examination of DEGs by applying the filter of *p* value < 0.05 and FDR < 0.05 sorted mainly metabolic pathways such as retinol metabolism (hsa00830), biosynthesis and metabolism of amino acids (hsa01230), antibiotics (hsa01130), and drug metabolism-cytochrome P450 (hsa00982) (Table 2). Some additional signaling pathways, i.e., chemical carcinogenesis (hsa05204), cell cycle (hsa04110), and p53 signaling pathway (hsa04115), were also markedly enriched by DEGs. The complete list of GO terms and KEGG pathways is enlisted in Supplementary Table 2.

### 2.3. STRING PPI Network Analysis and Interrelation between Pathways

**Figure 2:**
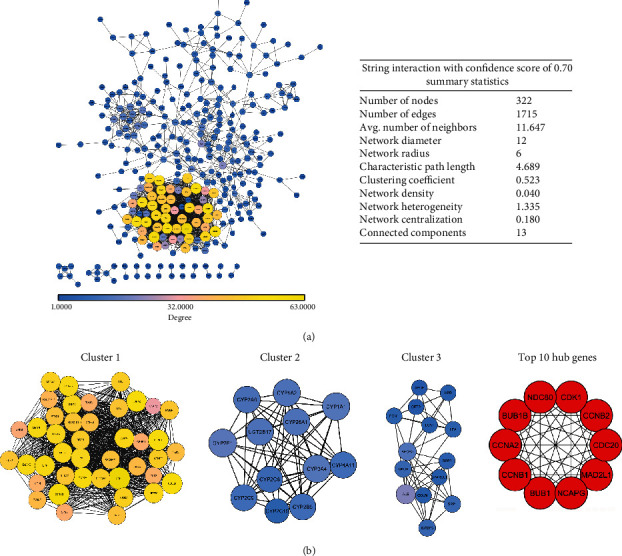
Protein-protein interaction and protein cluster formation. (a) Using the online STRING tool, a PPI network was developed which was further visualized by Cytoscape software. The size and color map nodes are determined by the degree value, which renders a gradual setting in small size with low degree in blue, large size with a high degree in yellow. (b) Top clusters determined by MCODE with score > 4 and node > 4. The top 10 genes derived from the MMC method were chosen using the CytoHubba plugin.

For a better understanding the role of the DEGs in HCC development, we constructed coexpression protein networks. The insertion of DEGs list into STRING and application of confidence score of >0.70 established a PPI network containing 1715 edges and 322 nodes, with each node connected with 11.6 other proteins on average. Different topological parameters for PPI network included network density of 0.040, network heterogeneity of 1.335, network centralization of 0.180, clustering coefficient of 0.523, and characteristic path length of 4.689. A complete interaction network is shown in Figure 2(a), in which degree and betweenness of topological features were calculated to distribute genes into different size circles and colors. The higher the value of these quantitative terms, the greater the importance in the network [41]. Through this PPI network, top clusters were sorted using Cytoscape plugin MCODE with score > 4 and nodes > 4. Cluster 1 was the densest interaction network showing MCODE score of 40.53, followed by cluster 2 and cluster 3 with scores 10.72 and 8.0, respectively (Figure 2(b)). Subsequently, several crucial hub genes including cyclin-dependent kinase (CDK1), cyclins (CCNA2, CCNB1, and CCNB2), serine/threonine-protein kinase (BUB1), NDC80, BUB1B, NCAPG, MAD2L1, and CDC20 were determined by CytoHubba plugin (Figure 2(b)). These selected hub genes were either involved in the cell cycle and its regulation or the main components of kinetochore-microtubule interaction.

**Figure 3:**
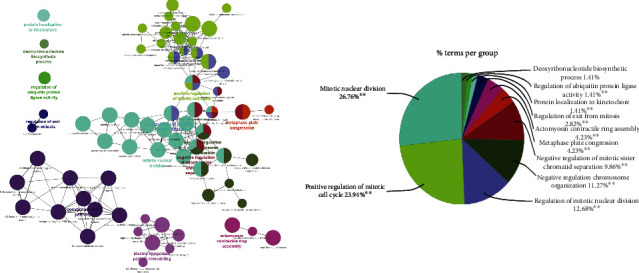
GO analysis of 10 hub genes mapped into 11 different biological processes. The group information and percentage are represented by different colors. Major portion of percent was mapped in mitotic nuclear division, positive regulation of mitotic cell cycle, regulation of mitotic nuclear division, negative regulation of chromosome organization, and negative regulation of mitotic sister chromatid separation.

Protein clusters were processed through ClueGO plugin of Cytoscape which suggested that cluster genes were mostly associated with nuclear and cellular division during meiosis and mitosis, chromosome reorganization, deoxyribonucleotide biosynthetic processes, and regulation of ubiquitin-protein ligase activity (Figure 3).

### 2.4. Survival Analysis through GEPIA and Expression Level of Hub Genes

**Figure 4:**
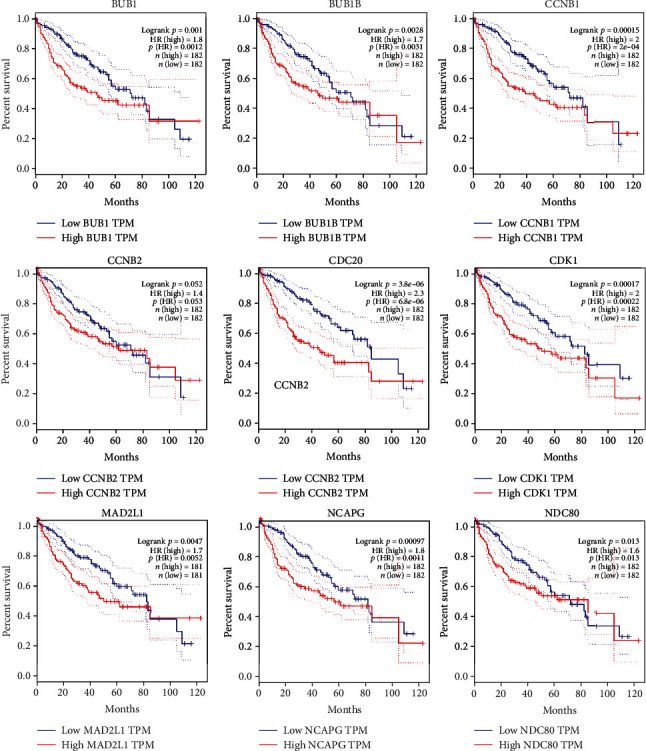
Kaplan-Meier survival analysis curve of the hub genes expressed in the HCC. The survival curves were plotted using the GEPIA web server. The gene candidates with high expression in the cohorts are shown in red, and the blue line indicates the low-expression cohort; the survival curve is represented in a dotted line, whereas the solid line is the 95% confidence interval. The logrank *p* value represents the overall significance of analysis, and HR stands for hazard ration; patient number (*n*) = 182.

**Figure 5:**
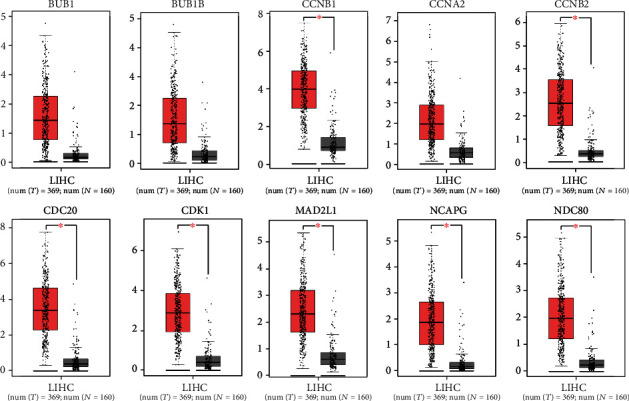
Gene expression analysis based on TCGA and GTEx data in GEPIA. The expression level of hub genes was validated in 369 LIHC (liver hepatocellular carcinoma) patients and 160 healthy controls. Interestingly, all hub genes were remarkably overexpressed in LIHC patients.

GEPIA servers provide a platform for integrated information of gene expression from TCGA and GTEx databases regarding multiple cancer types [42]. To evaluate the overall prognostic importance of hub genes in this study, Kaplan-Meier survival analysis was performed to examine the association between different expression levels of genes and survival time of patients with HCC. The logrank *p* value was estimated, where *p* value smaller than 0.05 indicates a statistically significant difference in mortality between the high-level and low-level groups. These high and low-level groups of patients were separated based on the median level of expression. The high expression level of CCNB2 (logrank *p* = 0.052) and NDC80 (logrank *p* = 0.013) demonstrated poor prognosis, while HCC patients showing high level of BUB1 (logrank *p* = 0.001), CDK1 (logrank *p* = 0.00017), NCAPG (logrank *p* = 0.00097), BUB1B (logrank *p* = 0.0028), CCNB1 (logrank *p* = 0.00015), CDC20 (logrank *p* = 3.8*e* − 06), and MAD2L1 (logrank *p* = 0.0047) had a higher risk of mortality (Figure 4). Furthermore, GEPIA boxplot representation of hub genes expression in 369 HCC patients and 160 normal/healthy persons described considerable increase in the level for all hub genes (Figure 5). These hub genes with high expression and low survival rate in patients suggested their association with the pathophysiology of liver cancer to varying extents, and these could be potential biomarkers for HCC prognosis to monitor the severity of liver cancer or a therapeutic target.

## 3. Discussion

Over recent years, tumor initiation and progression in HCC have been extensively researched; still, early diagnosis of HCC is truly a big challenge, because the exact mechanism of the induction of HCC needs a full understanding. Also, incidence and cancer-specific mortality worldwide are increasing due to limited therapeutic strategies; therefore, there is an urgent need to identify the potential key genes and mechanisms to precisely predict the HCC onset and progression and to develop novel therapeutic agents. Bioinformatics has become increasingly popular to evaluate changes in the gene expression profiles during the initiation and progression of diseases by the integrated microarray analysis, it helps to investigate the screened hub genes for cancer diagnosis and therapy.

In this study, we identified a total of 598 DGEs comprising 233 upregulated and 365 downregulated genes between HCC patients in comparison to healthy ones chosen from the GSE62232 expression profile datasets. Using the DAVID software, GO functional, and KEGG pathway enrichment analyses of the DEGs were performed. The results revealed that the identified DEGs were closely related to various biological processes and pathways such as metabolic, heme binding, drug detoxification, cell cycle, meiosis, and mitosis. We had also screened out ten hub genes, including cell cycle regulatory cyclins and cyclin-dependent proteins CCNA2, CCNB1, and CDK1. Besides, a PPI network was constructed to analyse the interactional relationships between the DGEs, and survival analyses of hub genes were performed using the GEPIA. These hub genes have been extensively researched in recent years.

Recent genetic evidence has revealed interphase cyclin-dependent kinases (CDKs) are essential for the proliferation of tumor cells. CDK1 belongs to the CDK family, a member of the Ser/Thr protein kinases necessary for cell-cycle progression, triggering cell cycle transitions, namely, G1/S and G2/M [43]. Clinical implications of deregulated CDK1 are closely related to HCC tumorigenesis. Research has found that overexpression of CDK1 in HCC is significantly negatively correlated with HCC patients' survival. Zhang et al. [44] suggested that miR-582-5p regulated the progression of HCC by directly targeting the CDK1. Elevated levels of CDK1 were also shown to be directly associated with advanced stage portal vein invasion, increased AFP levels, and poor patient survival in HCC [45]. Wu et al. [43] revealed higher levels of CDK1 in HCC patients than healthy individuals, which was in agreement with the present study's findings.

CCNA2, CCNB1, and CCNB2 genes are all members of the highly conserved cyclin family. In this study, high expression of CCNA2 was closely associated with poor prognosis in HCC patients. CCNA2 protein functions as regulators of the cell cycle by activating cyclin-dependent kinases, and CCNA2 expression in the cell cycle was driven mostly by E2Fs [46]. CCNA2 overexpression has been observed in several types of cancers; also, a study has demonstrated that inhibition of CCNA2 led to the suppression of HCC, cell proliferation, and tumorigenesis [47]. The aberrant expression of CCNA2 is related to reduce survival in patients with HCC and breast cancer [46, 48]. Chai et al. [49] revealed that CCNB1 is highly expressed and associated with the unfavorable prognosis for patients with HCC, consistent with our findings. This suggested the plausibility of CCNB1 as a potential therapeutic target for the treatment of HCC. This conclusion is further proved by a study that CCNB1 knockdown by miR-144 inhibited HCC cell migration, invasion, and proliferation [50]. Previous research [51] has shown that CCNB1 inhibits the growth of cells by inducing cell cycle arrest at the G2/M phase suggesting CCNB1 may be an effective anticancer agent in future therapy. Furthermore, CCNB2 was found to be overexpressed in several malignant tumors, and high expression of CCNB2 is associated with poor prognosis in HCC and invasive breast carcinoma [52, 53].

In the case of BUB1, several studies have found high expression of the BUB1 in a variety of human tumors. Serine/threonine-protein kinase BUB1 binds centromeres during mitosis [54] and has been involved in apoptosis and cell cycle [55], as well as in reducing the overall survival (OS) rate of HCC patients. BUB1B was reported to be involved in tumor cell cycle regulation, and overexpression of BUB1B is related to the progression and recurrence of HCC [56]. NDC80 is highly conserved, a core component of the kinetochore-microtubule interaction machinery which is identified as a requirement for proper chromosome segregation. NDC80 has also been associated with the HCC progression [57], and NDC80-knockdown in pancreatic cancer inhibited cell cycle and cell proliferation [58]. NCAPG organizes the coiling topology of individual chromatids. Liu et al. [59] demonstrated NCAPG functioning as an oncogene in the development of HCC.

CDC20 is an essential cell-cycle regulator, which plays an important role in promoting the onset of anaphase and mitotic exit. The increased expression levels of CDC20 have been linked with the development and progression of HCC [60]. Additionally, research has shown that silencing CDC20 and HPSE expression activated cell apoptosis; thus, targeting inhibition of both CDC20 and HPSE expression is an ideal therapeutic option of HCC [61]. As for MAD2L1, Yun et al. [62] demonstrated that MiR-200c-5p inhibits HCC cell proliferation, migration, and invasion by targeted suppression of MAD2L1, suggesting that the high expression levels of MAD2L1 are associated with poor prognosis of patients with HCC. Moreover, MAD2L1 may potentially be used as a prognostic and therapeutic target in HCC patients.

## 4. Material and Methods

### 4.1. Data Collection and Expression Profiling of DEGs

GEO contains datasets of gene expression, miRNA, and methylation patterns of diseased and healthy/control patients [63]. This study was focused on the expression profiling of HCC patients in comparison to healthy ones. A total of 91 samples were collected, including 81 HCC samples and 10 healthy samples as control [64]. GSE62232 was analysed through GEO2R [43] to evaluate and recalculate genes expressed uniquely in healthy and unhealthy samples. Analysis provided a lot of genes whose expression differed in both samples. To minimize the background noise, statistical filters of *p* value and fold-change value were used.

### 4.2. Functional Enrichment Annotation of DEGs

Functional enrichment analysis was performed to extract rational information from a set of DEGs. This provided us with the most prominent pathways being affected by the change of gene expression in diseased samples. The database named Database for Annotation, Visualization, and Integrated Discovery (DAVID; http://david.ncifcrf.gov) has been used to perform functional enrichment analysis. DAVID annotates the provided set of genes into the most affected processes such as Biological Processes (BP), Molecular Functions (MF), and Cellular Components (CC) [65]. We used GO and KEGG datasets in DAVID to annotate the most affected processes. KEGG is a database of molecular pathways containing detailed information about functional and biological systems [66]. The *p* value filtering ≤ 0.05 was used to exclude the results with a low confidence level.

### 4.3. Protein-Protein Interaction Network Backbone Analysis

Search Tool for the Retrieval of Interacting Genes (STRING; http://string-db.org) has been used to establish PPI network [67]. A confidence score of ≥0.07 was used to scrutinize statistically significant results. The PPIs network was visualized using Cytoscape (open-source platform), in which the most considerable module with a Molecular Complex Detection (MCODE) score of >5 and node score-cut-off of 0.02 was scanned with the Cytoscape plug-in MCODE.

### 4.4. Hub Gene Identification, Expression, and Survival Analysis

CytoHubba is a Cytoscape module that has been used in the PPI network for identifying primary hub genes. The MCC method was used to calculate the top 10 genes according to their importance in the system because it is comparatively recent and is highly recommended [68]. These top 10 genes having the highest MCC score were considered as hub genes. However, the Gene Expression Profiling Interactive Analysis (GEPIA) server provided a correlation of gene expression and their effect on the survival chances in specific cancer types [42]. Hub genes were analysed using GEPIA to calculate the impact of their differential expression on cancer progression.

### 4.5. Functional Annotation of MCODE Cluster Genes

ClueGO plugin was utilized to functionally annotate the top 3 MCODE Cluster genes into the biological process. It classified gene products into crucial biological processes using GO datasets as the reference [69]. This paper is published as preprint previously with the title “Identification of Novel Potential Biomarkers in Hepatocarcinoma Cancer; A Transcriptome Analysis” [70].

## 5. Conclusion

In summary, the purpose of the present study was to screen and verify hub genes that may provide new insights into the development, prognosis, and treatment of HCC. In total, 598 DEGs were identified via integrated bioinformatics analysis, of which ten were identified as hub genes that may be used as biomarkers for the diagnostic and prognostic evaluation of HCC. However, because the results of this study were based on data analysis only, further experimental verification via animal experiments and clinical trials are required to confirm these results.

## Figures and Tables

**Table 1:** Top 10 GO terms regarding biological process, molecular function, and cellular components after applying *p* value < 0.05 and FDR < 0.05 filter.

GO terms	Count	*p* value	FDR
Biological process (BP)			
GO:0055114~oxidation-reduction process	60	9.88*E* − 16	1.78*E* − 12
GO:0019373~epoxygenase P450 pathway	12	5.33*E* − 13	9.45*E* − 10
GO:0042738~exogenous drug catabolic process	8	1.70*E* − 08	3.01*E* − 05
GO:0008202~steroid metabolic process	12	4.80*E* − 08	8.52*E* − 05
GO:0017144~drug metabolic process	10	6.41*E* − 08	1.14*E* − 04
GO:0006805~xenobiotic metabolic process	15	9.23*E* − 08	1.64*E* − 04
GO:0097267~omega-hydroxylase P450 pathway	6	3.03*E* − 06	0.00537
GO:0006569~tryptophan catabolic process	6	3.03*E* − 06	0.00537
GO:0042493~response to drug	26	8.00*E* − 06	0.0142
GO:0051301~cell division	28	1.13*E* − 05	0.02011
Molecular function (MF)			
GO:0019825~oxygen binding	16	3.59*E* − 12	5.58*E* − 09
GO:0005506~iron ion binding	25	2.89*E* − 11	4.50*E* − 08
GO:0016705~oxidoreductase activity, acting on paired donors, with incorporation or reduction of molecular oxygen	16	7.99*E* − 11	1.24*E* − 07
GO:0004497~monooxygenase activity	16	1.05*E* − 10	1.63*E* − 07
GO:0020037~heme binding	22	7.83*E* − 10	1.22*E* − 06
GO:0008392~arachidonic acid epoxygenase activity	9	3.30*E* − 09	5.14*E* − 06
GO:0008395~steroid hydroxylase activity	10	3.61*E* − 08	5.62*E* − 05
GO:0016491~oxidoreductase activity	23	1.56*E* − 07	2.43*E* − 04
GO:0070330~aromatase activity	9	8.28*E* − 07	0.00129
GO:0009055~electron carrier activity	14	2.70*E* − 06	0.0042
GO:0016712~oxidoreductase activity, acting on paired donors, with incorporation or reduction of molecular oxygen, reduced flavin or flavoprotein as one donor, and incorporation of one atom of oxygen	7	2.80*E* − 06	0.00436
GO:0015171~amino acid transmembrane transporter activity	10	8.99*E* − 06	0.01399
Cellular components (CC)			
GO:0070062~extracellular exosome	152	1.98*E* − 14	2.72*E* − 11
GO:0031090~organelle membrane	21	6.51*E* − 13	8.96*E* − 10
GO:0005615~extracellular space	80	2.68*E* − 09	3.68*E* − 06
GO:0000777~condensed chromosome kinetochore	13	9.00*E* − 06	0.01238
GO:0072562~blood microparticle	17	1.10*E* − 05	0.01514
GO:0005829~cytosol	138	1.16*E* − 05	0.01594
GO:0005576~extracellular region	78	1.45*E* − 05	0.01989
GO:0030496~midbody	15	2.75*E* − 05	0.03775

**Table 2:** Top 10 pathways in which most of the DEGs enriched at *p* value < 0.05 and FDR < 0.05 filter.

Pathways ids	Pathways	*p* value	FDR
hsa01100	Metabolic pathways	9.19*E* − 10	1.19*E* − 06
hsa05204	Chemical carcinogenesis	6.68*E* − 08	8.63*E* − 05
hsa00830	Retinol metabolism	1.39*E* − 07	1.79*E* − 04
hsa04110	Cell cycle	3.38*E* − 07	4.37*E* − 04
hsa04115	p53 signalling pathway	1.69*E* − 06	0.002182762
hsa01130	Biosynthesis of antibiotics	2.94*E* − 06	0.003797444
hsa00980	Metabolism of xenobiotics by cytochrome P450	5.43*E* − 06	0.007016019
hsa03320	PPAR signalling pathway	1.02*E* − 05	0.01318125
hsa00982	Drug metabolism - cytochrome P450	1.20*E* − 05	0.01544763
hsa00260	Glycine, serine and threonine metabolism	1.59*E* − 05	0.020548859
hsa01230	Biosynthesis of amino acids	2.19*E* − 05	0.028277953

## Data Availability

We collected raw data of GSE62232 from https://www.ncbi.nlm.nih.gov/geo/query/acc.cgi?acc=GSE62232. It is an online repository, and data can be accessed directly from the study.

## Supplementary Materials

Supplementary Materials Supplementary Table 1: overall, 598 genes out of a total 19982 were differentially expressed in HCC samples with 233 upregulated and 365 downregulated genes.
