# Supplementary material for: Identification of Differentially Expressed Genes Associated with the Prognosis and Diagnosis of Hepatocellular Carcinoma by Integrated Bioinformatics Analysis
Source: Biomed Res Int. 2022 Oct 22;2022:4237633. doi: 10.1155/2022/4237633 (PMC9617698; doi:10.1155/2022/4237633)
Supplement: Supplementary Materials — Supplementary Table 1: overall, 598 genes out of a total 19982 were differentially expressed in HCC samples with 233 upregulated and 365 downregulated genes. [file 4237633.f1.docx]

**Supplementary Table 1:** Overall, 598 genes out of a total 19982 were differentially expressed in HCC samples with 233 upregulated and 365 downregulated genes

| S.No | Term | Pathways | *P*-value | FDR |
| --- | --- | --- | --- | --- |
| 1 | hsa01100 | Metabolic pathways | 9.19E-10 | 1.19E-06 |
| 2 | hsa05204 | Chemical carcinogenesis | 6.68E-08 | 8.63E-05 |
| 3 | hsa00830 | Retinol metabolism | 1.39E-07 | 1.79E-04 |
| 4 | hsa04110 | Cell cycle | 3.38E-07 | 4.37E-04 |
| 5 | hsa04115 | p53 signaling pathway | 1.69E-06 | 0.00218 |
| 6 | hsa01130 | Biosynthesis of antibiotics | 2.94E-06 | 0.0038 |
| 7 | hsa00980 | Metabolism of xenobiotics by cytochrome P450 | 5.43E-06 | 0.00702 |
| 8 | hsa03320 | PPAR signaling pathway | 1.02E-05 | 0.01318 |
| 9 | hsa00982 | Drug metabolism - cytochrome P450 | 1.20E-05 | 0.01545 |
| 10 | hsa00260 | Glycine, serine and threonine metabolism | 1.59E-05 | 0.02055 |
| 11 | hsa01230 | Biosynthesis of amino acids | 2.19E-05 | 0.02828 |

| S # | Gene symbol | adj.*P*.Val | *P*.Value | log^10p^ | t | B | up/  down | log^FC^ | Gene title |
| --- | --- | --- | --- | --- | --- | --- | --- | --- | --- |
| 1 | AKR1B10 | 0.0000141 | 0.000000405 | 6.392544977 | 5.461567 | 6.1661268 | up | 4.9225141 | aldoketo reductase family 1 member B10 |
| 2 | SPINK1 | 0.00157 | 0.000228 | 3.642065153 | 3.838524 | 0.1587785 | up | 4.1992338 | serine peptidase inhibitor, Kazal type 1 |
| 3 | GPC3 | 0.0000617 | 0.00000295 | 5.530177984 | 4.983981 | 4.2697642 | up | 4.1882414 | glypican 3 |
| 4 | DDX3Y | 0.00000334 | 5.96E-08 | 7.22475374 | 5.905418 | 8.0043543 | up | 4.1633333 | DEAD-box helicase 3, Y-linked |
| 5 | UBD///GABBR1 | 0.00000288 | 0.000000048 | 7.318758763 | 5.954778 | 8.2127583 | up | 4.0488713 | ubiquitin D///gamma-aminobutyric acid type B receptor subunit 1 |
| 6 | RPS4Y1 | 0.0000135 | 0.000000383 | 6.416801226 | 5.474833 | 6.2200722 | up | 4.0128604 | ribosomal protein S4, Y-linked 1 |
| 7 | COL15A1 | 2.34E-08 | 1.15E-10 | 9.93930216 | 7.278683 | 14.0310293 | up | 3.9732899 | collagen type XV alpha 1 chain |
| 8 | CCL20 | 0.0000459 | 0.00000199 | 5.701146924 | 5.080469 | 4.6453665 | up | 3.6720266 | C-C motif chemokine ligand 20 |
| 9 | ASPM | 7.04E-10 | 1.7E-12 | 11.76955108 | 8.167136 | 18.1019335 | up | 3.6385659 | abnormal spindle microtubule assembly |
| 10 | ACSL4 | 0.000336 | 0.0000287 | 4.542118103 | 4.405066 | 2.1062781 | up | 3.4698985 | acyl-CoA synthetase long-chain family member 4 |
| 11 | RRM2 | 8.01E-10 | 2.01E-12 | 11.69680394 | 8.13234 | 17.9410259 | up | 3.2621159 | ribonucleotide reductase regulatory subunit M2 |
| 12 | CRNDE | 0.0000432 | 0.00000183 | 5.73754891 | 5.10009 | 4.7222331 | up | 3.1100787 | colorectal neoplasia differentially expressed (non-protein coding) |
| 13 | TOP2A | 0.000000658 | 7.03E-09 | 8.153044675 | 6.385576 | 10.0613114 | up | 3.0157837 | topoisomerase (DNA) II alpha |
| 14 | RACGAP1 | 2.38E-10 | 4.53E-13 | 12.3439018 | 8.442791 | 19.3795571 | up | 2.940438 | Rac GTPase activating protein 1 |
| 15 | CAP2 | 3.86E-10 | 8.12E-13 | 12.09044397 | 8.321331 | 18.8160242 | up | 2.9330637 | CAP, adenylate cyclase-associated protein, 2 (yeast) |
| 16 | CDKN2C | 3.46E-11 | 4.87E-14 | 13.31247104 | 8.906126 | 21.5353083 | up | 2.9212475 | cyclin dependent kinase inhibitor 2C |
| 17 | ZWINT | 9.93E-10 | 2.58E-12 | 11.58838029 | 8.079955 | 17.6989475 | up | 2.9174163 | ZW10 interacting kinetochore protein |
| 18 | ANLN | 0.00000134 | 1.76E-08 | 7.754487332 | 6.181392 | 9.1787844 | up | 2.8773878 | anillin actin binding protein |
| 19 | CTHRC1 | 0.000564 | 0.0000574 | 4.241088108 | 4.220723 | 1.4527982 | up | 2.8524457 | collagen triple helix repeat containing 1 |
| 20 | SPP1 | 0.0119 | 0.0032 | 2.494850022 | 3.028596 | -2.2781047 | up | 2.8306263 | secreted phosphoprotein 1 |
| 21 | IL32 | 0.00000118 | 1.48E-08 | 7.829738285 | 6.219392 | 9.3421928 | up | 2.8143372 | interleukin 32 |
| 22 | MAD2L1 | 3.24E-08 | 1.67E-10 | 9.777283529 | 7.197935 | 13.6660139 | up | 2.6468978 | MAD2 mitotic arrest deficient-like 1 (yeast) |
| 23 | HKDC1 | 0.0000423 | 0.00000178 | 5.749579998 | 5.106758 | 4.7483894 | up | 2.6440169 | hexokinase domain containing 1 |
| 24 | PRC1 | 3.85E-08 | 2.06E-10 | 9.68613278 | 7.153274 | 13.4645727 | up | 2.6052807 | protein regulator of cytokinesis 1 |
| 25 | ECT2 | 1.59E-09 | 4.66E-12 | 11.33161408 | 7.955781 | 17.1260372 | up | 2.6045571 | epithelial cell transforming 2 |
| 26 | KRT23 | 0.0104 | 0.00269 | 2.57024772 | 3.086043 | -2.1201713 | up | 2.5992372 | keratin 23 |
| 27 | KIAA0101 | 7.21E-09 | 2.77E-11 | 10.55752023 | 7.580789 | 15.4050645 | up | 2.5864358 | KIAA0101 |
| 28 | ROBO1 | 0.000453 | 0.0000426 | 4.370590401 | 4.300626 | 1.7338002 | up | 2.5836553 | roundabout guidance receptor 1 |
| 29 | DUXAP10 | 0.000806 | 0.0000938 | 4.027797162 | 4.087268 | 0.991359 | up | 2.564569 | double homeobox A pseudogene 10 |
| 30 | FAT1 | 0.0000031 | 5.33E-08 | 7.273272791 | 5.930784 | 8.1113567 | up | 2.5230297 | FAT atypical cadherin 1 |
| 31 | GINS1 | 0.00000449 | 0.00000009 | 7.045757491 | 5.811149 | 7.6084464 | up | 2.510512 | GINS complex subunit 1 |
| 32 | COL1A2 | 0.00102 | 0.000127 | 3.896196279 | 4.002397 | 0.7031864 | up | 2.4985271 | collagen type I alpha 2 chain |
| 33 | CENPU | 0.000000217 | 1.7E-09 | 8.769551079 | 6.698141 | 11.4321052 | up | 2.4768743 | centromere protein U |
| 34 | PRKAA2 | 0.00095 | 0.000117 | 3.931814138 | 4.026654 | 0.7851228 | up | 2.4666019 | protein kinase AMP-activated catalytic subunit alpha 2 |
| 35 | PBK | 0.00000268 | 4.36E-08 | 7.360513511 | 5.976311 | 8.3039034 | up | 2.465334 | PDZ binding kinase |
| 36 | PRR11 | 0.00000134 | 1.76E-08 | 7.754487332 | 6.181201 | 9.1779652 | up | 2.4549923 | proline rich 11 |
| 37 | TMEM45B | 0.00477 | 0.000982 | 3.007888512 | 3.406005 | -1.1975737 | up | 2.4510078 | transmembrane protein 45B |
| 38 | CDKN3 | 0.0000171 | 0.000000522 | 6.282329497 | 5.401459 | 5.9225086 | up | 2.4467466 | cyclin dependent kinase inhibitor 3 |
| 39 | LCN2 | 0.00402 | 0.000785 | 3.105130343 | 3.474493 | -0.9909192 | up | 2.4286143 | lipocalin 2 |
| 40 | GPX2 | 0.0025 | 0.000422 | 3.374687549 | 3.660054 | -0.4154441 | up | 2.4181435 | glutathione peroxidase 2 |
| 41 | EIF1AY | 0.000222 | 0.0000163 | 4.787812396 | 4.552223 | 2.6407551 | up | 2.3922122 | eukaryotic translation initiation factor 1A, Y-linked |
| 42 | NDC80 | 0.000000915 | 1.08E-08 | 7.966576245 | 6.290625 | 9.6495572 | up | 2.3724942 | NDC80, kinetochore complex component |
| 43 | NEK2 | 0.00000738 | 0.000000173 | 6.761953897 | 5.660639 | 6.9823131 | up | 2.3613008 | NIMA related kinase 2 |
| 44 | CHI3L1 | 0.0505 | 0.0206 | 1.68613278 | 2.355694 | -3.9407308 | up | 2.3523952 | chitinase 3 like 1 |
| 45 | AURKA | 0.000000876 | 1.01E-08 | 7.995678626 | 6.305237 | 9.7127702 | up | 2.3379592 | aurora kinase A |
| 46 | TXLNGY | 0.000385 | 0.0000343 | 4.46470588 | 4.358512 | 1.939533 | up | 2.331997 | taxilin gamma pseudogene, Y-linked |
| 47 | EDIL3 | 0.000206 | 0.0000148 | 4.829738285 | 4.578135 | 2.7360052 | up | 2.3286427 | EGF like repeats and discoidin domains 3 |
| 48 | FKBP1B | 0.0014 | 0.000197 | 3.705533774 | 3.880674 | 0.2972665 | up | 2.3241895 | FK506 binding protein 1B |
| 49 | DTNA | 0.000102 | 0.00000577 | 5.238824187 | 4.817009 | 3.6294739 | up | 2.3063337 | dystrobrevin alpha |
| 50 | FABP5 | 0.000000243 | 1.99E-09 | 8.701146924 | 6.66339 | 11.2786065 | up | 2.3046967 | fatty acid binding protein 5 |
| 51 | FAM72A///FAM72D///FAM72B///FAM72C | 0.00000727 | 0.000000169 | 6.772113295 | 5.666184 | 7.0052461 | up | 2.2997606 | family with sequence similarity 72 member A///family with sequence similarity 72 member D///family with sequence similarity 72 member B///family with sequence similarity 72 member C |
| 52 | PLXNC1 | 0.000385 | 0.0000343 | 4.46470588 | 4.358501 | 1.9394936 | up | 2.2584931 | plexin C1 |
| 53 | SULT1C2 | 0.00561 | 0.00121 | 2.91721463 | 3.340502 | -1.3922489 | up | 2.2517633 | sulfotransferase family 1C member 2 |
| 54 | LYZ | 0.0241 | 0.00802 | 2.095825632 | 2.710949 | -3.1069573 | up | 2.2398918 | lysozyme |
| 55 | LEF1 | 0.000769 | 0.0000881 | 4.055024092 | 4.104436 | 1.0501572 | up | 2.2365608 | lymphoid enhancer binding factor 1 |
| 56 | TOX3 | 0.00172 | 0.000257 | 3.590066877 | 3.80416 | 0.0466732 | up | 2.2341123 | TOX high mobility group box family member 3 |
| 57 | FGF13 | 0.000777 | 0.0000895 | 4.048176965 | 4.100013 | 1.0349916 | up | 2.2232277 | fibroblast growth factor 13 |
| 58 | XK | 0.000881 | 0.000106 | 3.974694135 | 4.054341 | 0.8790636 | up | 2.2175393 | X-linked Kx blood group |
| 59 | DHRS2 | 0.0655 | 0.0286 | 1.543633967 | 2.2248 | -4.2219722 | up | 2.2096946 | dehydrogenase/reductase 2 |
| 60 | PLVAP | 1.7E-10 | 3.05E-13 | 12.51570016 | 8.525105 | 19.7619163 | up | 2.1893751 | plasmalemma vesicle associated protein |
| 61 | CCNA2 | 0.000000184 | 1.38E-09 | 8.860120914 | 6.743094 | 11.6310491 | up | 2.1790249 | cyclin A2 |
| 62 | ZIC2 | 0.00178 | 0.000271 | 3.567030709 | 3.789503 | -0.0009191 | up | 2.1640978 | Zic family member 2 |
| 63 | DLGAP5 | 0.0000199 | 0.000000648 | 6.188424994 | 5.350413 | 5.7166898 | up | 2.1608257 | DLG associated protein 5 |
| 64 | KIF20A | 0.00000196 | 0.000000029 | 7.537602002 | 6.068698 | 8.696533 | up | 2.1432886 | kinesin family member 20A |
| 65 | KDM5D | 0.0000969 | 0.00000539 | 5.268411235 | 4.834086 | 3.6943803 | up | 2.1394925 | lysine demethylase 5D |
| 66 | FLVCR1 | 0.0000162 | 0.000000485 | 6.314258261 | 5.419261 | 5.9945215 | up | 2.134648 | feline leukemia virus subgroup C cellular receptor 1 |
| 67 | LOC101930489///MIR4435-2HG///LINC00152 | 0.000000494 | 4.88E-09 | 8.311580178 | 6.46605 | 10.4120542 | up | 2.1309558 | uncharacterized LOC101930489///MIR4435-2 host gene///long intergenic non-protein coding RNA 152 |
| 68 | UHRF1 | 0.000178 | 0.0000121 | 4.91721463 | 4.628704 | 2.9228516 | up | 2.1245332 | ubiquitin like with PHD and ring finger domains 1 |
| 69 | GOLM1 | 0.00302 | 0.000541 | 3.266802735 | 3.586536 | -0.6461263 | up | 2.1200895 | golgi membrane protein 1 |
| 70 | VWF | 0.000000821 | 9.31E-09 | 8.031050319 | 6.323307 | 9.7910242 | up | 2.1099379 | von Willebrand factor |
| 71 | CDK1 | 0.0000382 | 0.00000156 | 5.806875402 | 5.139185 | 4.8758687 | up | 2.1017497 | cyclin dependent kinase 1 |
| 72 | UBE2T | 0.0000032 | 5.57E-08 | 7.254144805 | 5.920832 | 8.0693531 | up | 2.0783535 | ubiquitin conjugating enzyme E2 T |
| 73 | BIRC5 | 0.0000111 | 0.000000296 | 6.528708289 | 5.535535 | 6.4677427 | up | 2.0770844 | baculoviral IAP repeat containing 5 |
| 74 | APOBEC3B | 0.00014 | 0.00000872 | 5.059483515 | 4.712982 | 3.2370202 | up | 2.0417107 | apolipoprotein B mRNA editing enzyme catalytic subunit 3B |
| 75 | FAM83D | 0.0000153 | 0.000000448 | 6.348721986 | 5.437674 | 6.069129 | up | 2.038122 | family with sequence similarity 83 member D |
| 76 | TP53I3 | 0.000000202 | 1.54E-09 | 8.812479279 | 6.718807 | 11.523511 | up | 2.0377711 | tumor protein p53 inducible protein 3 |
| 77 | NCAPG | 0.0000929 | 0.00000509 | 5.293282218 | 4.848635 | 3.7497852 | up | 2.0339777 | non-SMC condensin I complex subunit G |
| 78 | TMCO3 | 0.000000121 | 8.21E-10 | 9.085656843 | 6.855738 | 12.1313972 | up | 2.0293625 | transmembrane and coiled-coil domains 3 |
| 79 | TTK | 0.0000685 | 0.00000338 | 5.4710833 | 4.950273 | 4.1394992 | up | 2.015594 | TTK protein kinase |
| 80 | SLC38A1 | 0.00633 | 0.00142 | 2.847711656 | 3.290867 | -1.5378061 | up | 2.0058654 | solute carrier family 38 member 1 |
| 81 | BUB1B | 0.0000118 | 0.000000322 | 6.492144128 | 5.515629 | 6.3863785 | up | 2.0026994 | BUB1 mitotic checkpoint serine/threonine kinase B |
| 82 | TRIM16 | 0.00184 | 0.000281 | 3.55129368 | 3.778438 | -0.0367622 | up | 1.9991748 | tripartite motif containing 16 |
| 83 | KDELR3 | 0.000231 | 0.0000172 | 4.764471553 | 4.538764 | 2.5914121 | up | 1.982206 | KDEL endoplasmic reticulum protein retention receptor 3 |
| 84 | ANXA2 | 1.31E-10 | 2.27E-13 | 12.64397414 | 8.586556 | 20.0475723 | up | 1.9818987 | annexin A2 |
| 85 | SFN | 0.00259 | 0.000443 | 3.353596274 | 3.645945 | -0.4599838 | up | 1.9755603 | stratifin |
| 86 | EZH2 | 0.00000652 | 0.000000146 | 6.835647144 | 5.700056 | 7.1455634 | up | 1.9633624 | enhancer of zeste 2 polycomb repressive complex 2 subunit |
| 87 | CENPF | 0.0000094 | 0.000000236 | 6.627087997 | 5.58785 | 6.6822525 | up | 1.9630484 | centromere protein F |
| 88 | LAPTM4B | 0.000147 | 0.00000935 | 5.029188389 | 4.69538 | 3.171122 | up | 1.9613851 | lysosomal protein transmembrane 4 beta |
| 89 | CENPK | 0.0000273 | 0.000000973 | 6.01188716 | 5.253188 | 5.3274607 | up | 1.9570109 | centromere protein K |
| 90 | NMRAL1P1 | 0.00222 | 0.000359 | 3.444905551 | 3.707142 | -0.2658785 | up | 1.9470643 | NmrA like redox sensor 1 pseudogene 1 |
| 91 | CKS2 | 0.00000347 | 6.32E-08 | 7.199282922 | 5.89226 | 7.9489242 | up | 1.927907 | CDC28 protein kinase regulatory subunit 2 |
| 92 | NUF2 | 0.000887 | 0.000107 | 3.970616222 | 4.051607 | 0.8697664 | up | 1.9225813 | NUF2, NDC80 kinetochore complex component |
| 93 | NUSAP1 | 0.00000327 | 5.76E-08 | 7.239577517 | 5.913082 | 8.0366627 | up | 1.921678 | nucleolar and spindle associated protein 1 |
| 94 | SOX9 | 0.0347 | 0.0128 | 1.89279003 | 2.539738 | -3.5214238 | up | 1.9192926 | SRY-box 9 |
| 95 | PDZK1IP1 | 0.0164 | 0.00488 | 2.311580178 | 2.885224 | -2.6616466 | up | 1.9106471 | PDZK1 interacting protein 1 |
| 96 | NIPAL2 | 0.0000494 | 0.0000022 | 5.657577319 | 5.055894 | 4.5493226 | up | 1.898436 | NIPA like domain containing 2 |
| 97 | DNAJC6 | 0.000239 | 0.0000181 | 4.742321425 | 4.526473 | 2.5464339 | up | 1.8868134 | DnaJ heat shock protein family (Hsp40) member C6 |
| 98 | MAP2 | 0.00619 | 0.00138 | 2.860120914 | 3.299879 | -1.5115057 | up | 1.8719142 | microtubule associated protein 2 |
| 99 | HOXA13 | 0.00809 | 0.00195 | 2.709965389 | 3.190476 | -1.8269607 | up | 1.8685393 | homeobox A13 |
| 100 | CDC20 | 0.0000279 | 0.000001 | 6 | 5.245967 | 5.2987017 | up | 1.8676522 | cell division cycle 20 |
| 101 | STXBP6 | 0.000479 | 0.0000462 | 4.335358024 | 4.279237 | 1.6582388 | up | 1.8573667 | syntaxin binding protein 6 |
| 102 | RBP7 | 0.000000854 | 9.78E-09 | 8.009661145 | 6.312345 | 9.7435424 | up | 1.8385232 | retinol binding protein 7 |
| 103 | ITGA6 | 0.000000426 | 4.03E-09 | 8.394694954 | 6.508539 | 10.5978696 | up | 1.8250398 | integrin subunit alpha 6 |
| 104 | RBM24 | 0.0043 | 0.000858 | 3.066512712 | 3.447542 | -1.0726163 | up | 1.823753 | RNA binding motif protein 24 |
| 105 | PSPH | 0.00000881 | 0.000000217 | 6.663540266 | 5.607384 | 6.7625997 | up | 1.8228361 | phosphoserine phosphatase |
| 106 | PDGFA | 0.0000171 | 0.000000527 | 6.278189385 | 5.399274 | 5.9136787 | up | 1.8224222 | platelet derived growth factor subunit A |
| 107 | SQLE | 0.000524 | 0.0000522 | 4.282329497 | 4.246652 | 1.5436031 | up | 1.8193159 | squalene epoxidase |
| 108 | BUB1 | 0.0000548 | 0.00000254 | 5.595166283 | 5.02071 | 4.4122686 | up | 1.8152182 | BUB1 mitotic checkpoint serine/threonine kinase |
| 109 | RFX5 | 6.11E-11 | 9.73E-14 | 13.01188716 | 8.762656 | 20.8670022 | up | 1.8129367 | regulatory factor X5 |
| 110 | CXCL10 | 0.00319 | 0.000582 | 3.235077015 | 3.564905 | -0.7133329 | up | 1.8127696 | C-X-C motif chemokine ligand 10 |
| 111 | TKT | 0.0000213 | 0.000000705 | 6.151810883 | 5.330145 | 5.6352457 | up | 1.8067453 | transketolase |
| 112 | PTTG1 | 0.000000509 | 5.05E-09 | 8.296708622 | 6.458754 | 10.3801899 | up | 1.8064966 | pituitary tumor-transforming 1 |
| 113 | PODXL | 0.00000308 | 5.28E-08 | 7.277366077 | 5.93296 | 8.1205478 | up | 1.8063253 | podocalyxin like |
| 114 | IQGAP3 | 0.000158 | 0.0000103 | 4.987162775 | 4.669466 | 3.0743754 | up | 1.802944 | IQ motif containing GTPase activating protein 3 |
| 115 | COCH | 0.0277 | 0.00959 | 2.018181393 | 2.646274 | -3.2662397 | up | 1.8000103 | cochlin |
| 116 | SUCO | 1.92E-08 | 9.04E-11 | 10.04383157 | 7.329584 | 14.2616329 | up | 1.7982811 | SUN domain containing ossification factor |
| 117 | GAS2L3 | 0.0000164 | 0.000000494 | 6.306273051 | 5.414904 | 5.9768868 | up | 1.7961977 | growth arrest specific 2 like 3 |
| 118 | TOMM40L | 3.26E-10 | 6.68E-13 | 12.17522354 | 8.362004 | 19.0046393 | up | 1.7943582 | translocase of outer mitochondrial membrane 40 like |
| 119 | SLC38A6 | 0.00000005 | 2.88E-10 | 9.540607512 | 7.081521 | 13.1416304 | up | 1.7920759 | solute carrier family 38 member 6 |
| 120 | CD200 | 0.0000273 | 0.000000972 | 6.012333735 | 5.253334 | 5.3280427 | up | 1.7888444 | CD200 molecule |
| 121 | CBFA2T2 | 0.000000206 | 1.59E-09 | 8.798602876 | 6.711815 | 11.4925766 | up | 1.7862521 | CBFA2/RUNX1 translocation partner 2 |
| 122 | LGALS3 | 0.00333 | 0.000614 | 3.211831629 | 3.548556 | -0.7639259 | up | 1.7790315 | lectin, galactoside binding soluble 3 |
| 123 | MCM6 | 0.00000128 | 1.66E-08 | 7.779891912 | 6.193926 | 9.2326426 | up | 1.7788486 | minichromosome maintenance complex component 6 |
| 124 | PHLDA2 | 0.00115 | 0.00015 | 3.823908741 | 3.956115 | 0.5478092 | up | 1.7751412 | pleckstrin homology like domain family A member 2 |
| 125 | SLC39A10 | 0.00000479 | 9.81E-08 | 7.008330993 | 5.791434 | 7.5260045 | up | 1.7733303 | solute carrier family 39 member 10 |
| 126 | S100P | 0.0809 | 0.0375 | 1.425968732 | 2.111632 | -4.4535023 | up | 1.7670563 | S100 calcium binding protein P |
| 127 | SLC7A11 | 0.0108 | 0.00285 | 2.54515514 | 3.066443 | -2.1743256 | up | 1.765208 | solute carrier family 7 member 11 |
| 128 | GORAB | 0.000000707 | 7.71E-09 | 8.112945622 | 6.365094 | 9.9722991 | up | 1.7651419 | golgin, RAB6 interacting |
| 129 | GMNN | 0.00000991 | 0.000000252 | 6.598599459 | 5.572613 | 6.6196785 | up | 1.7619864 | geminin, DNA replication inhibitor |
| 130 | TYMS | 0.0000108 | 0.000000284 | 6.54668166 | 5.544665 | 6.5051094 | up | 1.7468327 | thymidylate synthetase |
| 131 | EBF1 | 0.0000058 | 0.000000127 | 6.896196279 | 5.732352 | 7.279706 | up | 1.740387 | early B-cell factor 1 |
| 132 | PTGFRN | 0.0000373 | 0.0000015 | 5.823908741 | 5.148034 | 4.9107306 | up | 1.740352 | prostaglandin F2 receptor inhibitor |
| 133 | ACKR3 | 0.000694 | 0.0000766 | 4.11577123 | 4.142634 | 1.181577 | up | 1.736647 | atypical chemokine receptor 3 |
| 134 | HSP90AB1 | 0.000109 | 0.00000629 | 5.201349355 | 4.795344 | 3.5473245 | up | 1.7339352 | heat shock protein 90 alpha family class B member 1 |
| 135 | SGO2 | 0.0000351 | 0.00000139 | 5.8569852 | 5.167937 | 4.9892605 | up | 1.7314311 | shugoshin 2 |
| 136 | ERICH5 | 0.0145 | 0.00414 | 2.382999659 | 2.941125 | -2.513922 | up | 1.7249539 | glutamate rich 5 |
| 137 | CCNB2 | 0.0000233 | 0.000000795 | 6.099632871 | 5.301615 | 5.5208698 | up | 1.7240497 | cyclin B2 |
| 138 | F2RL1 | 0.0334 | 0.0122 | 1.913640169 | 2.557779 | -3.4788461 | up | 1.7210165 | F2R like trypsin receptor 1 |
| 139 | ZFY | 0.00015 | 0.00000957 | 5.019088062 | 4.689406 | 3.1487881 | up | 1.7164173 | zinc finger protein, Y-linked |
| 140 | HMGN4 | 9.9E-12 | 1.19E-14 | 13.92445304 | 9.197481 | 22.8936072 | up | 1.7163931 | high mobility group nucleosomal binding domain 4 |
| 141 | CCNB1 | 0.000304 | 0.0000252 | 4.598599459 | 4.439336 | 2.2297529 | up | 1.7142967 | cyclin B1 |
| 142 | MIR452///MIR224///GABRE | 0.00271 | 0.000471 | 3.326979093 | 3.62773 | -0.517298 | up | 1.7099506 | microRNA 452///microRNA 224///gamma-aminobutyric acid type A receptor epsilon subunit |
| 143 | MELK | 0.0000349 | 0.00000138 | 5.860120914 | 5.169617 | 4.995898 | up | 1.6994439 | maternal embryonic leucine zipper kinase |
| 144 | DTL | 0.000673 | 0.000073 | 4.13667714 | 4.155767 | 1.2269524 | up | 1.6982277 | denticleless E3 ubiquitin protein ligase homolog |
| 145 | ERAP2 | 0.0273 | 0.00941 | 2.026410377 | 2.653121 | -3.2495333 | up | 1.6947457 | endoplasmic reticulum aminopeptidase 2 |
| 146 | CDH11 | 0.014 | 0.00397 | 2.401209493 | 2.955326 | -2.4760217 | up | 1.6942672 | cadherin 11 |
| 147 | UPK3BL///POLR2J3///LOC441259///POLR2J2///POLR2J | 0.00000479 | 9.76E-08 | 7.010550182 | 5.792516 | 7.5305286 | up | 1.6866418 | uroplakin 3B-like///RNA polymerase II subunit J3///PMS1 homolog 2, mismatch repair system component pseudogene///RNA polymerase II subunit J2///RNA polymerase II subunit J |
| 148 | TDGF1P3///TDGF1 | 0.043 | 0.0168 | 1.774690718 | 2.435701 | -3.7618415 | up | 1.6863301 | teratocarcinoma-derived growth factor 1 pseudogene 3///teratocarcinoma-derived growth factor 1 |
| 149 | SPARCL1 | 0.00348 | 0.000651 | 3.186419011 | 3.531256 | -0.817274 | up | 1.6856511 | SPARC like 1 |
| 150 | LRRC1 | 0.000395 | 0.0000355 | 4.449771647 | 4.349593 | 1.907717 | up | 1.6838134 | leucine rich repeat containing 1 |
| 151 | GNAL | 0.00677 | 0.00155 | 2.809668302 | 3.263092 | -1.6185146 | up | 1.677955 | G protein subunit alpha L |
| 152 | FABP4 | 0.00548 | 0.00118 | 2.928117993 | 3.349667 | -1.3651887 | up | 1.6741423 | fatty acid binding protein 4 |
| 153 | CA12 | 0.0214 | 0.00685 | 2.164309429 | 2.767112 | -2.9659946 | up | 1.6739511 | carbonic anhydrase 12 |
| 154 | CD109 | 0.0125 | 0.0034 | 2.468521083 | 3.007816 | -2.3346379 | up | 1.6734239 | CD109 molecule |
| 155 | CENPW | 0.00000549 | 0.000000118 | 6.928117993 | 5.748305 | 7.3460975 | up | 1.6732048 | centromere protein W |
| 156 | GJC1 | 0.0000103 | 0.000000265 | 6.576754126 | 5.560987 | 6.5719833 | up | 1.6715134 | gap junction protein gamma 1 |
| 157 | CDKN2B | 0.0000625 | 0.000003 | 5.522878745 | 4.979894 | 4.2539447 | up | 1.6697341 | cyclin dependent kinase inhibitor 2B |
| 158 | SPATS2 | 0.000000163 | 1.2E-09 | 8.920818754 | 6.772853 | 11.7629851 | up | 1.6642161 | spermatogenesis associated serine rich 2 |
| 159 | RNF213 | 0.00000138 | 1.84E-08 | 7.735182177 | 6.170622 | 9.1325425 | up | 1.6633699 | ring finger protein 213 |
| 160 | RAP2A | 3.48E-08 | 1.82E-10 | 9.739928612 | 7.180068 | 13.5853873 | up | 1.6619235 | RAP2A, member of RAS oncogene family |
| 161 | CCDC34 | 0.0000038 | 7.13E-08 | 7.14691047 | 5.864588 | 7.8325347 | up | 1.6611458 | coiled-coil domain containing 34 |
| 162 | HIST1H2BJ///HIST1H2BG | 0.00343 | 0.00064 | 3.193820026 | 3.536472 | -0.8012106 | up | 1.6507607 | histone cluster 1, H2bj///histone cluster 1, H2bg |
| 163 | FAM169A | 0.00585 | 0.00128 | 2.89279003 | 3.32279 | -1.4443872 | up | 1.6419674 | family with sequence similarity 169 member A |
| 164 | COL1A1 | 0.0164 | 0.00488 | 2.311580178 | 2.884948 | -2.6623703 | up | 1.6413932 | collagen type I alpha 1 chain |
| 165 | MYO5A | 0.0000265 | 0.000000934 | 6.029653124 | 5.26297 | 5.3664564 | up | 1.6407369 | myosin VA |
| 166 | PLA2G4C | 0.0000611 | 0.00000291 | 5.536107011 | 4.98701 | 4.2814932 | up | 1.6393242 | phospholipase A2 group IVC |
| 167 | NQO1 | 0.0521 | 0.0214 | 1.669586227 | 2.340622 | -3.9738421 | up | 1.6311593 | NAD(P)H quinone dehydrogenase 1 |
| 168 | STC1 | 0.000103 | 0.00000587 | 5.231361899 | 4.812601 | 3.612741 | up | 1.6308705 | stanniocalcin 1 |
| 169 | PCDH17 | 0.0000645 | 0.00000313 | 5.504455662 | 4.969179 | 4.2125013 | up | 1.6285858 | protocadherin 17 |
| 170 | HELLS | 0.000336 | 0.0000287 | 4.542118103 | 4.405481 | 2.1077688 | up | 1.6275166 | helicase, lymphoid-specific |
| 171 | PIEZO2 | 0.0000101 | 0.000000258 | 6.588380294 | 5.567021 | 6.5967315 | up | 1.6269991 | piezo type mechanosensitive ion channel component 2 |
| 172 | LGALS4 | 0.0883 | 0.0419 | 1.377785977 | 2.063557 | -4.5485486 | up | 1.6235755 | galectin 4 |
| 173 | BCAT1 | 0.000175 | 0.0000118 | 4.928117993 | 4.636232 | 2.9507717 | up | 1.6202205 | branched chain amino acid transaminase 1 |
| 174 | SCD | 0.00389 | 0.000752 | 3.123782159 | 3.487648 | -0.9508657 | up | 1.6182349 | stearoyl-CoA desaturase |
| 175 | C15orf48 | 0.0412 | 0.0159 | 1.798602876 | 2.456713 | -3.7139917 | up | 1.617574 | chromosome 15 open reading frame 48 |
| 176 | HSPB1 | 0.000000132 | 9.29E-10 | 9.031984286 | 6.828979 | 12.0123093 | up | 1.6164423 | heat shock protein family B (small) member 1 |
| 177 | KIAA1462 | 0.0000209 | 0.000000689 | 6.161780778 | 5.335591 | 5.657116 | up | 1.6120823 | KIAA1462 |
| 178 | KIF14 | 0.000795 | 0.0000922 | 4.035269079 | 4.091974 | 1.0074594 | up | 1.610892 | kinesin family member 14 |
| 179 | ME1 | 0.0158 | 0.00466 | 2.331614083 | 2.901231 | -2.6195872 | up | 1.6107085 | malic enzyme 1 |
| 180 | TMEM267 | 0.000000013 | 5.5E-11 | 10.25963731 | 7.435288 | 14.7417319 | up | 1.6094795 | transmembrane protein 267 |
| 181 | EPPK1 | 0.0381 | 0.0144 | 1.841637508 | 2.495301 | -3.6251826 | up | 1.6058016 | epiplakin 1 |
| 182 | UBE2S | 0.0000182 | 0.00000057 | 6.244125144 | 5.380578 | 5.8381973 | up | 1.5990541 | ubiquitin conjugating enzyme E2 S |
| 183 | IGF2BP3 | 0.0378 | 0.0143 | 1.844663963 | 2.498382 | -3.6180415 | up | 1.5959094 | insulin like growth factor 2 mRNA binding protein 3 |
| 184 | SLC26A2 | 0.00000391 | 0.000000074 | 7.13076828 | 5.855898 | 7.796032 | up | 1.595023 | solute carrier family 26 member 2 |
| 185 | LOC100130872///SPON2 | 0.000171 | 0.0000114 | 4.943095149 | 4.643846 | 2.9790442 | up | 1.5908268 | uncharacterized LOC100130872///spondin 2 |
| 186 | MDK | 0.000984 | 0.000122 | 3.913640169 | 4.013894 | 0.7419777 | up | 1.5905169 | midkine (neurite growth-promoting factor 2) |
| 187 | GBP2 | 0.00143 | 0.000201 | 3.696803943 | 3.874257 | 0.2761121 | up | 1.5902581 | guanylate binding protein 2 |
| 188 | RARRES1 | 0.0168 | 0.00505 | 2.296708622 | 2.873444 | -2.6924757 | up | 1.5881414 | retinoic acid receptor responder 1 |
| 189 | CDKN2A | 0.000401 | 0.0000363 | 4.440093375 | 4.343446 | 1.8858139 | up | 1.5878739 | cyclin dependent kinase inhibitor 2A |
| 190 | CD24 | 0.0817 | 0.0379 | 1.42136079 | 2.106483 | -4.4637766 | up | 1.5868733 | CD24 molecule |
| 191 | HMMR | 0.0000234 | 0.000000797 | 6.098541679 | 5.300977 | 5.5183191 | up | 1.584629 | hyaluronan mediated motility receptor |
| 192 | CCNE2 | 0.00159 | 0.000232 | 3.634512015 | 3.833989 | 0.1439424 | up | 1.5764747 | cyclin E2 |
| 193 | ANXA2P2 | 7.8E-09 | 3.01E-11 | 10.5214335 | 7.562898 | 15.3233526 | up | 1.5757711 | annexin A2 pseudogene 2 |
| 194 | RASEF | 0.0241 | 0.00801 | 2.096367484 | 2.71122 | -3.1062826 | up | 1.5745544 | RAS and EF-hand domain containing |
| 195 | GTSE1 | 0.0000923 | 0.00000504 | 5.297569464 | 4.8508 | 3.7580378 | up | 1.5740922 | G2 and S-phase expressed 1 |
| 196 | RAD51AP1 | 0.0000936 | 0.00000513 | 5.289882635 | 4.846284 | 3.7408249 | up | 1.5736771 | RAD51 associated protein 1 |
| 197 | COL4A2 | 0.000269 | 0.0000213 | 4.671620397 | 4.483481 | 2.3897032 | up | 1.5733515 | collagen type IV alpha 2 chain |
| 198 | STK39 | 0.00571 | 0.00124 | 2.906578315 | 3.333026 | -1.4142815 | up | 1.5635159 | serine/threonine kinase 39 |
| 199 | IRAK1 | 0.000000252 | 2.08E-09 | 8.681936665 | 6.653369 | 11.2343901 | up | 1.5618569 | interleukin 1 receptor associated kinase 1 |
| 200 | MALAT1 | 0.0000854 | 0.00000453 | 5.343901798 | 4.877415 | 3.8596626 | up | 1.5600663 | metastasis associated lung adenocarcinoma transcript 1 (non-protein coding) |
| 201 | DLG5 | 0.0000162 | 0.000000483 | 6.316052869 | 5.420001 | 5.9975153 | up | 1.5597538 | discs large MAGUK scaffold protein 5 |
| 202 | ATAD2 | 0.000619 | 0.0000653 | 4.185086819 | 4.185898 | 1.3314238 | up | 1.5560837 | ATPase family, AAA domain containing 2 |
| 203 | FKBP11 | 0.0000095 | 0.00000024 | 6.619788758 | 5.584753 | 6.6695296 | up | 1.5520979 | FK506 binding protein 11 |
| 204 | USP9Y | 0.0043 | 0.000857 | 3.067019178 | 3.447752 | -1.071982 | up | 1.54909 | ubiquitin specific peptidase 9, Y-linked |
| 205 | PAQR5 | 0.0323 | 0.0117 | 1.931814138 | 2.574128 | -3.4400368 | up | 1.5484811 | progestin and adipoQ receptor family member 5 |
| 206 | KIF4A | 0.000464 | 0.0000443 | 4.353596274 | 4.290384 | 1.6975874 | up | 1.5444849 | kinesin family member 4A |
| 207 | LOC101928195///LOC100996643///MTHFD1L | 0.0000381 | 0.00000155 | 5.809668302 | 5.140436 | 4.8807939 | up | 1.5421222 | methylenetetrahydrofolate dehydrogenase (NADP+ dependent) 1-like pseudogene///monofunctional C1-tetrahydrofolate synthase, mitochondrial-like///methylenetetrahydrofolate dehydrogenase (NADP+ dependent) 1-like |
| 208 | MFSD6 | 0.000251 | 0.0000194 | 4.71219827 | 4.507941 | 2.4787576 | up | 1.5405681 | major facilitator superfamily domain containing 6 |
| 209 | E2F3 | 0.00000217 | 3.31E-08 | 7.480172006 | 6.039055 | 8.5702809 | up | 1.5369175 | E2F transcription factor 3 |
| 210 | MTMR11 | 0.00374 | 0.000713 | 3.14691047 | 3.503619 | -0.9020843 | up | 1.5358646 | myotubularin related protein 11 |
| 211 | DLEU2 | 0.000000922 | 1.09E-08 | 7.962573502 | 6.287651 | 9.6366991 | up | 1.5344188 | deleted in lymphocytic leukemia 2 (non-protein coding) |
| 212 | TTC39A | 0.00324 | 0.000594 | 3.226213555 | 3.558703 | -0.7325481 | up | 1.5339825 | tetratricopeptide repeat domain 39A |
| 213 | MAGEA1 | 0.0491 | 0.0199 | 1.701146924 | 2.370746 | -3.9074775 | up | 1.5306342 | MAGE family member A1 |
| 214 | SLC1A4 | 0.0000338 | 0.00000131 | 5.882728704 | 5.182483 | 5.0467585 | up | 1.5300911 | solute carrier family 1 member 4 |
| 215 | HMGB2 | 0.00000816 | 0.000000198 | 6.70333481 | 5.629317 | 6.8529677 | up | 1.5279635 | high mobility group box 2 |
| 216 | C1orf112 | 0.0000461 | 0.000002 | 5.698970004 | 5.078567 | 4.6379255 | up | 1.5250718 | chromosome 1 open reading frame 112 |
| 217 | SLC44A3 | 0.00412 | 0.00081 | 3.091514981 | 3.464907 | -1.020034 | up | 1.5231004 | solute carrier family 44 member 3 |
| 218 | MCM3 | 0.000000922 | 1.09E-08 | 7.962573502 | 6.287436 | 9.6357683 | up | 1.5226885 | minichromosome maintenance complex component 3 |
| 219 | CKAP2 | 0.00000153 | 2.11E-08 | 7.675717545 | 6.140641 | 9.0039859 | up | 1.5183513 | cytoskeleton associated protein 2 |
| 220 | LOC101927345///LOC101060632///LOC101059949///ANKRD20A12P | 0.0129 | 0.00356 | 2.448550002 | 2.992576 | -2.3758987 | up | 1.5166594 | putative ankyrin repeat domain-containing protein 20A12 pseudogene///uncharacterized gene LOC101060632///putative ankyrin repeat domain-containing protein 20A12 pseudogene///ankyrin repeat domain 20 family member A12, pseudogene |
| 221 | UBE2C | 0.000183 | 0.0000126 | 4.899629455 | 4.619357 | 2.8882209 | up | 1.5156906 | ubiquitin conjugating enzyme E2 C |
| 222 | NEB | 0.0286 | 0.01 | 2 | 2.630518 | -3.3045488 | up | 1.5153069 | nebulin |
| 223 | TXNRD1 | 0.000262 | 0.0000206 | 4.68613278 | 4.492808 | 2.4236259 | up | 1.5145306 | thioredoxin reductase 1 |
| 224 | COL4A1 | 0.000566 | 0.0000576 | 4.239577517 | 4.219797 | 1.4495644 | up | 1.5135284 | collagen type IV alpha 1 chain |
| 225 | SMARCA4 | 1.74E-09 | 5.35E-12 | 11.27164622 | 7.926967 | 16.9932888 | up | 1.5131214 | SWI/SNF related, matrix associated, actin dependent regulator of chromatin, subfamily a, member 4 |
| 226 | SMOC2 | 0.00978 | 0.00249 | 2.603800653 | 3.110545 | -2.0520837 | up | 1.5124707 | SPARC related modular calcium binding 2 |
| 227 | HCP5 | 0.0000517 | 0.00000234 | 5.630784143 | 5.040971 | 4.4911279 | up | 1.5062954 | HLA complex P5 (non-protein coding) |
| 228 | C12orf49 | 0.000725 | 0.0000813 | 4.089909454 | 4.126336 | 1.1254051 | up | 1.5058931 | chromosome 12 open reading frame 49 |
| 229 | TDRKH | 0.0000334 | 0.00000128 | 5.89279003 | 5.186344 | 5.0620359 | up | 1.5047719 | tudor and KH domain containing |
| 230 | ABCC4 | 0.000954 | 0.000117 | 3.931814138 | 4.025258 | 0.7803965 | up | 1.5041887 | ATP binding cassette subfamily C member 4 |
| 231 | ZNF207 | 0.00000342 | 6.18E-08 | 7.209011525 | 5.897251 | 7.9699448 | up | 1.5036064 | zinc finger protein 207 |
| 232 | IER5 | 0.00000422 | 0.000000082 | 7.086186148 | 5.832631 | 7.6984177 | up | 1.5018714 | immediate early response 5 |
| 233 | SOWAHA | 0.000661 | 0.0000711 | 4.148130399 | 4.162947 | 1.251804 | up | 1.5007689 | sosondowah ankyrin repeat domain family member A |
| 234 | CPS1 | 0.0916 | 0.044 | 1.356547324 | -2.042779 | -4.5890139 | down | -1.5016854 | carbamoyl-phosphate synthase 1 |
| 235 | PLEK2 | 0.0000852 | 0.00000451 | 5.345823458 | -4.878324 | 3.8631419 | down | -1.5028852 | pleckstrin 2 |
| 236 | ALDH6A1 | 0.000072 | 0.00000361 | 5.442492798 | -4.933955 | 4.0766212 | down | -1.5060617 | aldehyde dehydrogenase 6 family member A1 |
| 237 | PCK2 | 0.0000668 | 0.00000327 | 5.485452247 | -4.958357 | 4.1706945 | down | -1.5082421 | phosphoenolpyruvate carboxykinase 2, mitochondrial |
| 238 | SARDH | 0.00000046 | 4.45E-09 | 8.351639989 | -6.486741 | 10.5024894 | down | -1.5095833 | sarcosine dehydrogenase |
| 239 | TMEM45A | 0.0876 | 0.0415 | 1.381951903 | -2.067658 | -4.5405186 | down | -1.5110981 | transmembrane protein 45A |
| 240 | SOCS3 | 0.00759 | 0.0018 | 2.744727495 | -3.216282 | -1.7533106 | down | -1.5149471 | suppressor of cytokine signaling 3 |
| 241 | HS3ST3B1 | 0.00438 | 0.000878 | 3.056505484 | -3.440314 | -1.0944436 | down | -1.5151104 | heparan sulfate-glucosamine 3-sulfotransferase 3B1 |
| 242 | RCAN1 | 0.00000046 | 4.45E-09 | 8.351639989 | -6.486522 | 10.5015306 | down | -1.515651 | regulator of calcineurin 1 |
| 243 | GADD45B | 0.000104 | 0.00000593 | 5.226945307 | -4.810121 | 3.6033318 | down | -1.5159414 | growth arrest and DNA damage inducible beta |
| 244 | SLC39A5 | 0.00133 | 0.000183 | 3.73754891 | -3.901098 | 0.3647587 | down | -1.5168496 | solute carrier family 39 member 5 |
| 245 | ATOH8 | 0.00000273 | 4.47E-08 | 7.349692477 | -5.971153 | 8.2820595 | down | -1.51904 | atonal bHLH transcription factor 8 |
| 246 | C11orf96 | 0.00959 | 0.00243 | 2.614393726 | -3.118767 | -2.0291379 | down | -1.5192134 | chromosome 11 open reading frame 96 |
| 247 | NAMPT | 0.00296 | 0.000527 | 3.278189385 | -3.594474 | -0.6213855 | down | -1.5228665 | nicotinamide phosphoribosyltransferase |
| 248 | RCL1 | 0.00000663 | 0.000000149 | 6.826813732 | -5.695132 | 7.1251409 | down | -1.5248656 | RNA terminal phosphate cyclase like 1 |
| 249 | PDE7B | 0.000000433 | 4.11E-09 | 8.386158178 | -6.504133 | 10.57858 | down | -1.5276462 | phosphodiesterase 7B |
| 250 | NRG1 | 0.0146 | 0.0042 | 2.37675071 | -2.936807 | -2.5254162 | down | -1.5277401 | neuregulin 1 |
| 251 | ASPN | 0.0304 | 0.0108 | 1.966576245 | -2.602305 | -3.3726516 | down | -1.5308932 | asporin |
| 252 | ALB | 0.00131 | 0.000178 | 3.749579998 | -3.908408 | 0.3889759 | down | -1.5321971 | albumin |
| 253 | ART4 | 0.0215 | 0.0069 | 2.161150909 | -2.764519 | -2.9725551 | down | -1.5325477 | ADP-ribosyltransferase 4 (Dombrock blood group) |
| 254 | PRODH2 | 0.00039 | 0.0000349 | 4.457174573 | -4.353719 | 1.9224274 | down | -1.5326587 | proline dehydrogenase 2 |
| 255 | ARHGEF26 | 0.000712 | 0.0000794 | 4.100179498 | -4.132985 | 1.1483035 | down | -1.5369446 | Rho guanine nucleotide exchange factor 26 |
| 256 | PTPRD | 0.0166 | 0.00497 | 2.303643611 | -2.879168 | -2.67751 | down | -1.5414059 | protein tyrosine phosphatase, receptor type D |
| 257 | ACSL5 | 0.00442 | 0.000889 | 3.051098239 | -3.436713 | -1.1053067 | down | -1.5436017 | acyl-CoA synthetase long-chain family member 5 |
| 258 | ETNK2 | 0.000694 | 0.0000765 | 4.116338565 | -4.142898 | 1.1824895 | down | -1.5453774 | ethanolamine kinase 2 |
| 259 | BGN | 0.016 | 0.00471 | 2.326979093 | -2.897607 | -2.6291266 | down | -1.5511073 | biglycan |
| 260 | TSLP | 0.00566 | 0.00123 | 2.910094889 | -3.336349 | -1.4044932 | down | -1.5534779 | thymic stromal lymphopoietin |
| 261 | HPR | 0.0216 | 0.00696 | 2.15739076 | -2.761162 | -2.9810436 | down | -1.5548138 | haptoglobin-related protein |
| 262 | RNF165 | 3.95E-10 | 8.45E-13 | 12.07314329 | -8.313007 | 18.7774314 | down | -1.5558164 | ring finger protein 165 |
| 263 | PTGIS | 0.00757 | 0.00179 | 2.747146969 | -3.217577 | -1.7496005 | down | -1.5584964 | prostaglandin I2 (prostacyclin) synthase |
| 264 | TGFA | 0.000522 | 0.0000519 | 4.284832642 | -4.24777 | 1.5475267 | down | -1.5607577 | transforming growth factor alpha |
| 265 | ABCG2 | 0.0145 | 0.00414 | 2.382999659 | -2.941407 | -2.5131707 | down | -1.5613865 | ATP binding cassette subfamily G member 2 (Junior blood group) |
| 266 | RNA45S5 | 0.00824 | 0.00199 | 2.701146924 | -3.183003 | -1.8482022 | down | -1.5630354 | RNA, 45S pre-ribosomal 5 |
| 267 | SLC19A3 | 0.0000283 | 0.00000102 | 5.991399828 | -5.240985 | 5.2788696 | down | -1.5640132 | solute carrier family 19 member 3 |
| 268 | ENO3 | 0.00526 | 0.00111 | 2.954677021 | -3.366804 | -1.3144305 | down | -1.566151 | enolase 3 |
| 269 | SLC38A4 | 0.0164 | 0.00489 | 2.310691141 | -2.884352 | -2.6639312 | down | -1.5671706 | solute carrier family 38 member 4 |
| 270 | SAA2-SAA4///SAA4 | 0.0232 | 0.00764 | 2.116906641 | -2.728036 | -3.0643298 | down | -1.5690884 | SAA2-SAA4 readthrough///serum amyloid A4, constitutive |
| 271 | SKAP1 | 0.000963 | 0.000119 | 3.924453039 | -4.021559 | 0.7678836 | down | -1.5721046 | src kinase associated phosphoprotein 1 |
| 272 | CYP2C18 | 0.0311 | 0.0111 | 1.954677021 | -2.591562 | -3.3984185 | down | -1.5731701 | cytochrome P450 family 2 subfamily C member 18 |
| 273 | GPD1 | 0.000235 | 0.0000176 | 4.754487332 | -4.532802 | 2.5695858 | down | -1.5767808 | glycerol-3-phosphate dehydrogenase 1 |
| 274 | CA2 | 0.00478 | 0.000985 | 3.00656377 | -3.404921 | -1.2008193 | down | -1.5776012 | carbonic anhydrase 2 |
| 275 | HAAO | 0.00000608 | 0.000000134 | 6.872895202 | -5.719161 | 7.224874 | down | -1.5798157 | 3-hydroxyanthranilate 3,4-dioxygenase |
| 276 | PHYHD1 | 0.000584 | 0.0000602 | 4.220403509 | -4.208115 | 1.4087779 | down | -1.5801217 | phytanoyl-CoA dioxygenase domain containing 1 |
| 277 | IGHM | 0.00921 | 0.0023 | 2.638272164 | -3.136084 | -1.9806515 | down | -1.5837686 | immunoglobulin heavy constant mu |
| 278 | SAMD5 | 0.00417 | 0.000821 | 3.085656843 | -3.460729 | -1.0327044 | down | -1.589259 | sterile alpha motif domain containing 5 |
| 279 | LOC101927331 | 0.00578 | 0.00126 | 2.899629455 | -3.327788 | -1.4296967 | down | -1.5907675 | uncharacterized LOC101927331 |
| 280 | FAM149A | 0.00105 | 0.000133 | 3.876148359 | -3.990646 | 0.6636147 | down | -1.5910711 | family with sequence similarity 149 member A |
| 281 | OGDHL | 0.00608 | 0.00135 | 2.869666232 | -3.307477 | -1.4892869 | down | -1.5914783 | oxoglutarate dehydrogenase-like |
| 282 | PON1 | 0.00777 | 0.00185 | 2.732828272 | -3.206641 | -1.780881 | down | -1.593498 | paraoxonase 1 |
| 283 | THBS1 | 0.0115 | 0.00307 | 2.512861625 | -3.041984 | -2.241513 | down | -1.5978506 | thrombospondin 1 |
| 284 | NPC1L1 | 0.0235 | 0.00774 | 2.111259039 | -2.723682 | -3.0752124 | down | -1.6001821 | NPC1 like intracellular cholesterol transporter 1 |
| 285 | EHD3 | 4.66E-08 | 2.61E-10 | 9.583359493 | -7.102503 | 13.2359735 | down | -1.6069736 | EH domain containing 3 |
| 286 | EDNRB | 0.000726 | 0.0000815 | 4.088842391 | -4.125662 | 1.1230846 | down | -1.6090639 | endothelin receptor type B |
| 287 | ADH6 | 0.00593 | 0.0013 | 2.886056648 | -3.317783 | -1.459087 | down | -1.6094539 | alcohol dehydrogenase 6 (class V) |
| 288 | PLGLB1///PLGLB2 | 0.0241 | 0.008 | 2.096910013 | -2.711766 | -3.1049237 | down | -1.6195383 | plasminogen-like B1///plasminogen-like B2 |
| 289 | ACMSD | 0.00583 | 0.00128 | 2.89279003 | -3.324353 | -1.4397959 | down | -1.6222869 | aminocarboxymuconate semialdehyde decarboxylase |
| 290 | MASP2 | 0.00348 | 0.000651 | 3.186419011 | -3.531345 | -0.8169988 | down | -1.6278013 | mannan binding lectin serine peptidase 2 |
| 291 | SORL1 | 0.0000031 | 5.35E-08 | 7.271646218 | -5.929987 | 8.1079919 | down | -1.6289116 | sortilin related receptor 1 |
| 292 | SERPINA4 | 0.00155 | 0.000223 | 3.651695137 | -3.844592 | 0.1786467 | down | -1.6324992 | serpin family A member 4 |
| 293 | SMIM24 | 0.00161 | 0.000236 | 3.627087997 | -3.828896 | 0.1272944 | down | -1.6343265 | small integral membrane protein 24 |
| 294 | CFHR4 | 0.0273 | 0.00941 | 2.026410377 | -2.652866 | -3.2501552 | down | -1.6352191 | complement factor H related 4 |
| 295 | ARSE | 0.019 | 0.00589 | 2.229884705 | -2.819829 | -2.83147 | down | -1.6352629 | arylsulfatase E (chondrodysplasia punctata 1) |
| 296 | SLC27A5 | 0.00316 | 0.000573 | 3.241845378 | -3.569235 | -0.6999062 | down | -1.6382055 | solute carrier family 27 member 5 |
| 297 | ERRFI1 | 0.00275 | 0.000479 | 3.319664487 | -3.622668 | -0.5331873 | down | -1.6474664 | ERBB receptor feedback inhibitor 1 |
| 298 | EPHX2 | 0.000193 | 0.0000135 | 4.869666232 | -4.600702 | 2.81923 | down | -1.6493846 | epoxide hydrolase 2 |
| 299 | FOXO1 | 2.3E-09 | 7.46E-12 | 11.12726117 | -7.857282 | 16.6725709 | down | -1.6504365 | forkhead box O1 |
| 300 | ADAMTS13 | 1.39E-16 | 7.13E-20 | 19.14691047 | -11.709813 | 34.4894167 | down | -1.6515733 | ADAM metallopeptidase with thrombospondin type 1 motif 13 |
| 301 | RBP5 | 0.000277 | 0.0000223 | 4.651695137 | -4.471862 | 2.3475047 | down | -1.6597416 | retinol binding protein 5 |
| 302 | ADRB1 | 0.00294 | 0.000522 | 3.282329497 | -3.597163 | -0.6129979 | down | -1.6646497 | adrenoceptor beta 1 |
| 303 | MAT1A | 0.0000108 | 0.000000284 | 6.54668166 | -5.545011 | 6.5065261 | down | -1.6664588 | methionine adenosyltransferase 1A |
| 304 | ALAS1 | 0.0000138 | 0.000000393 | 6.40560745 | -5.468725 | 6.1952256 | down | -1.6683272 | 5'-aminolevulinate synthase 1 |
| 305 | F9 | 0.0115 | 0.00307 | 2.512861625 | -3.042052 | -2.2413278 | down | -1.6683497 | coagulation factor IX |
| 306 | NAAA | 0.000000381 | 3.56E-09 | 8.448550002 | -6.535593 | 10.7164083 | down | -1.6712912 | N-acylethanolamine acid amidase |
| 307 | FOXP2 | 0.000431 | 0.00004 | 4.397940009 | -4.317823 | 1.7947334 | down | -1.6714271 | forkhead box P2 |
| 308 | ANGPTL6 | 5.72E-17 | 2.61E-20 | 19.58335949 | -11.923195 | 35.4529579 | down | -1.6716055 | angiopoietin like 6 |
| 309 | MS4A6A | 0.0000543 | 0.0000025 | 5.602059991 | -5.024167 | 4.4257124 | down | -1.6717309 | membrane spanning 4-domains A6A |
| 310 | NCOR1 | 1.71E-13 | 1.5E-16 | 15.82390874 | -10.105061 | 27.1201988 | down | -1.6739695 | nuclear receptor corepressor 1 |
| 311 | MRC1 | 0.00254 | 0.000431 | 3.36552273 | -3.654085 | -0.4343035 | down | -1.6760355 | mannose receptor, C type 1 |
| 312 | GIPC2 | 0.00189 | 0.000292 | 3.534617149 | -3.76791 | -0.0707941 | down | -1.6777723 | GIPC PDZ domain containing family member 2 |
| 313 | DNAJC12 | 0.00252 | 0.000427 | 3.369572125 | -3.656615 | -0.4263128 | down | -1.6788109 | DnaJ heat shock protein family (Hsp40) member C12 |
| 314 | SLC1A1 | 0.0251 | 0.00843 | 2.074172425 | -2.693056 | -3.1513513 | down | -1.6797527 | solute carrier family 1 member 1 |
| 315 | ID2B///ID2 | 0.000179 | 0.0000122 | 4.913640169 | -4.627139 | 2.9170497 | down | -1.6815079 | inhibitor of DNA binding 2B, HLH protein (pseudogene)///inhibitor of DNA binding 2, HLH protein |
| 316 | ACOT12 | 0.00991 | 0.00254 | 2.595166283 | -3.104686 | -2.0684052 | down | -1.6816759 | acyl-CoA thioesterase 12 |
| 317 | NGFR | 8.62E-08 | 5.34E-10 | 9.272458743 | -6.948572 | 12.5456292 | down | -1.6817027 | nerve growth factor receptor |
| 318 | ACADSB | 0.0000162 | 0.000000486 | 6.313363731 | -5.418472 | 5.9913255 | down | -1.6833715 | acyl-CoA dehydrogenase, short/branched chain |
| 319 | ACVR1C | 0.00000189 | 2.76E-08 | 7.559090918 | -6.07983 | 8.7440096 | down | -1.6867222 | activin A receptor type 1C |
| 320 | ACAA1 | 0.000000915 | 1.08E-08 | 7.966576245 | -6.29085 | 9.6505308 | down | -1.6868499 | acetyl-CoA acyltransferase 1 |
| 321 | AADACP1 | 0.000941 | 0.000115 | 3.93930216 | -4.030587 | 0.79844 | down | -1.6894693 | arylacetamide deacetylase pseudogene 1 |
| 322 | TSKU | 0.000251 | 0.0000194 | 4.71219827 | -4.507922 | 2.4786895 | down | -1.6919954 | tsukushi, small leucine rich proteoglycan |
| 323 | FAM151A | 0.00000347 | 6.33E-08 | 7.19859629 | -5.891807 | 7.9470193 | down | -1.6921894 | family with sequence similarity 151 member A |
| 324 | PRKAR2B | 0.00011 | 0.00000637 | 5.195860568 | -4.792418 | 3.5362432 | down | -1.6925963 | protein kinase cAMP-dependent type II regulatory subunit beta |
| 325 | PPM1K | 0.000273 | 0.0000219 | 4.659555885 | -4.47661 | 2.3647407 | down | -1.6926753 | protein phosphatase, Mg2+/Mn2+ dependent 1K |
| 326 | ETFDH | 0.00000534 | 0.000000114 | 6.943095149 | -5.756965 | 7.3821725 | down | -1.694559 | electron transfer flavoprotein dehydrogenase |
| 327 | AASS | 0.0027 | 0.000468 | 3.329754147 | -3.629701 | -0.5111064 | down | -1.6981727 | aminoadipate-semialdehyde synthase |
| 328 | GCH1 | 0.0000354 | 0.0000014 | 5.853871964 | -5.165227 | 4.9785602 | down | -1.7003459 | GTP cyclohydrolase 1 |
| 329 | HAL | 0.0818 | 0.038 | 1.420216403 | -2.105941 | -4.4648557 | down | -1.7007091 | histidine ammonia-lyase |
| 330 | ST3GAL6 | 0.000427 | 0.0000396 | 4.402304814 | -4.320638 | 1.8047214 | down | -1.7045247 | ST3 beta-galactoside alpha-2,3-sialyltransferase 6 |
| 331 | TRPM8 | 0.00945 | 0.00238 | 2.623423043 | -3.1252 | -2.0111517 | down | -1.7103888 | transient receptor potential cation channel subfamily M member 8 |
| 332 | LOC149703 | 0.00135 | 0.000185 | 3.732828272 | -3.897525 | 0.3529319 | down | -1.7169756 | uncharacterized LOC149703 |
| 333 | JCHAIN | 0.0867 | 0.0409 | 1.388276692 | -2.074096 | -4.5278836 | down | -1.7208161 | joining chain of multimeric IgA and IgM |
| 334 | CD4 | 1.53E-09 | 4.34E-12 | 11.36251027 | -7.971077 | 17.1965375 | down | -1.721388 | CD4 molecule |
| 335 | DMGDH | 0.00794 | 0.0019 | 2.721246399 | -3.198222 | -1.8049036 | down | -1.7240476 | dimethylglycine dehydrogenase |
| 336 | NR1I2 | 0.00393 | 0.000761 | 3.118615343 | -3.483929 | -0.9622012 | down | -1.7279625 | nuclear receptor subfamily 1 group I member 2 |
| 337 | MRO | 9.72E-10 | 2.51E-12 | 11.60032628 | -8.085773 | 17.7258235 | down | -1.7318216 | maestro |
| 338 | GRAMD1C | 0.000221 | 0.0000163 | 4.787812396 | -4.553683 | 2.6461112 | down | -1.7361672 | GRAM domain containing 1C |
| 339 | HPD | 0.0474 | 0.019 | 1.721246399 | -2.388266 | -3.8685369 | down | -1.7366574 | 4-hydroxyphenylpyruvate dioxygenase |
| 340 | ACSM5 | 0.006 | 0.00132 | 2.879426069 | -3.312916 | -1.4733584 | down | -1.7385054 | acyl-CoA synthetase medium-chain family member 5 |
| 341 | CIDEB | 0.0000349 | 0.00000138 | 5.860120914 | -5.169866 | 4.9968799 | down | -1.7436552 | cell death-inducing DFFA-like effector b |
| 342 | SPG20 | 0.00000375 | 7.01E-08 | 7.154281982 | -5.868511 | 7.8490196 | down | -1.7469408 | spastic paraplegia 20 (Troyer syndrome) |
| 343 | FABP1 | 0.0785 | 0.036 | 1.443697499 | -2.127989 | -4.4207113 | down | -1.7506208 | fatty acid binding protein 1 |
| 344 | SLC46A3 | 0.00224 | 0.000364 | 3.438898616 | -3.703342 | -0.2780012 | down | -1.7549402 | solute carrier family 46 member 3 |
| 345 | CYP4X1 | 0.000207 | 0.0000149 | 4.826813732 | -4.576356 | 2.7294538 | down | -1.755689 | cytochrome P450 family 4 subfamily X member 1 |
| 346 | KANK4 | 0.000034 | 0.00000132 | 5.879426069 | -5.180109 | 5.0373707 | down | -1.7563195 | KN motif and ankyrin repeat domains 4 |
| 347 | TAT | 0.00516 | 0.00109 | 2.962573502 | -3.374679 | -1.291041 | down | -1.7669053 | tyrosine aminotransferase |
| 348 | ADIRF | 0.011 | 0.0029 | 2.537602002 | -3.060825 | -2.1897965 | down | -1.7756747 | adipogenesis regulatory factor |
| 349 | XDH | 0.00575 | 0.00125 | 2.903089987 | -3.33024 | -1.422482 | down | -1.7781079 | xanthine dehydrogenase |
| 350 | SRD5A1 | 0.000268 | 0.0000212 | 4.673664139 | -4.484493 | 2.3933814 | down | -1.7815566 | steroid 5 alpha-reductase 1 |
| 351 | IYD | 0.000239 | 0.0000181 | 4.742321425 | -4.526254 | 2.5456302 | down | -1.7825587 | iodotyrosine deiodinase |
| 352 | C8A | 0.00217 | 0.000349 | 3.457174573 | -3.7157 | -0.2385457 | down | -1.7864035 | complement component 8 alpha subunit |
| 353 | PCDH9 | 1.62E-09 | 4.76E-12 | 11.32239305 | -7.951505 | 17.1063314 | down | -1.7896251 | protocadherin 9 |
| 354 | AKR7A3 | 0.000057 | 0.00000267 | 5.573488739 | -5.008009 | 4.3629234 | down | -1.7946826 | aldo-keto reductase family 7 member A3 |
| 355 | GREM2 | 0.0162 | 0.0048 | 2.318758763 | -2.890777 | -2.6470767 | down | -1.7953939 | gremlin 2, DAN family BMP antagonist |
| 356 | EPHB1 | 4.09E-19 | 1.18E-22 | 21.92811799 | -13.087591 | 40.6234434 | down | -1.7958186 | EPH receptor B1 |
| 357 | EPB41L4B | 0.00000441 | 8.75E-08 | 7.057991947 | -5.817627 | 7.6355614 | down | -1.7987337 | erythrocyte membrane protein band 4.1 like 4B |
| 358 | ADRA1A | 0.000029 | 0.00000106 | 5.974694135 | -5.233737 | 5.2500376 | down | -1.7990119 | adrenoceptor alpha 1A |
| 359 | CFP | 4.58E-15 | 2.92E-18 | 17.53461715 | -10.92658 | 30.9165621 | down | -1.8035621 | complement factor properdin |
| 360 | MIR675///H19 | 0.0254 | 0.00859 | 2.066006836 | -2.686048 | -3.1686718 | down | -1.8132784 | microRNA 675///H19, imprinted maternally expressed transcript (non-protein coding) |
| 361 | DEPDC7 | 0.000211 | 0.0000153 | 4.815308569 | -4.569997 | 2.7060538 | down | -1.817869 | DEP domain containing 7 |
| 362 | C8orf4 | 0.000401 | 0.0000363 | 4.440093375 | -4.343618 | 1.8864254 | down | -1.8204249 | chromosome 8 open reading frame 4 |
| 363 | IDNK | 4.27E-10 | 9.29E-13 | 12.03198429 | -8.293292 | 18.6860543 | down | -1.8231326 | IDNK, gluconokinase |
| 364 | FCGR2B | 0.000299 | 0.0000246 | 4.609064893 | -4.445419 | 2.2517329 | down | -1.8252543 | Fc fragment of IgG receptor IIb |
| 365 | PGLYRP2 | 0.0143 | 0.00406 | 2.391473966 | -2.947713 | -2.4963591 | down | -1.8264149 | peptidoglycan recognition protein 2 |
| 366 | ACAA2 | 0.000000619 | 6.46E-09 | 8.189767482 | -6.404236 | 10.1425003 | down | -1.8308448 | acetyl-CoA acyltransferase 2 |
| 367 | ALDH8A1 | 0.00649 | 0.00147 | 2.832682665 | -3.280588 | -1.5677367 | down | -1.8324598 | aldehyde dehydrogenase 8 family member A1 |
| 368 | PBLD | 0.00147 | 0.000209 | 3.679853714 | -3.86317 | 0.2396235 | down | -1.8456342 | phenazine biosynthesis like protein domain containing |
| 369 | DACH1 | 3.18E-20 | 5.23E-24 | 23.28149831 | -13.775423 | 43.6009516 | down | -1.8481837 | dachshund family transcription factor 1 |
| 370 | LOC101927157///NIPAL1 | 0.000072 | 0.00000361 | 5.442492798 | -4.933493 | 4.07484 | down | -1.8490754 | uncharacterized LOC101927157///NIPA like domain containing 1 |
| 371 | C6 | 0.012 | 0.00326 | 2.4867824 | -3.022344 | -2.295146 | down | -1.8510771 | complement component 6 |
| 372 | LOC100505985 | 0.00535 | 0.00114 | 2.943095149 | -3.359996 | -1.3346206 | down | -1.8519319 | uncharacterized LOC100505985 |
| 373 | SDS | 0.0266 | 0.00912 | 2.040005162 | -2.664497 | -3.2216931 | down | -1.8529411 | serine dehydratase |
| 374 | CYP2E1 | 0.0429 | 0.0168 | 1.774690718 | -2.436907 | -3.7591053 | down | -1.8547534 | cytochrome P450 family 2 subfamily E member 1 |
| 375 | SLC6A12 | 0.0000759 | 0.0000039 | 5.408935393 | -4.914927 | 4.0034437 | down | -1.8651797 | solute carrier family 6 member 12 |
| 376 | COLEC10 | 6.97E-17 | 3.31E-20 | 19.48017201 | -11.872564 | 35.2247427 | down | -1.8662186 | collectin subfamily member 10 |
| 377 | VIPR1 | 2.34E-18 | 7.71E-22 | 21.11294562 | -12.67991 | 38.830891 | down | -1.8687309 | vasoactive intestinal peptide receptor 1 |
| 378 | ATF5 | 0.00935 | 0.00235 | 2.628932138 | -3.129938 | -1.9978836 | down | -1.8706897 | activating transcription factor 5 |
| 379 | PPID | 8.29E-09 | 3.25E-11 | 10.48811664 | -7.547011 | 15.2508307 | down | -1.8788019 | peptidylprolyl isomerase D |
| 380 | TFF3 | 0.0000833 | 0.00000437 | 5.359518563 | -4.886643 | 3.8949771 | down | -1.8813676 | trefoil factor 3 |
| 381 | ITGA9 | 0.000139 | 0.00000861 | 5.064996849 | -4.716144 | 3.2488727 | down | -1.8842554 | integrin subunit alpha 9 |
| 382 | PPARGC1A | 0.00146 | 0.000207 | 3.684029655 | -3.865718 | 0.2480017 | down | -1.8846991 | PPARG coactivator 1 alpha |
| 383 | LEPROT///LEPR | 0.000273 | 0.0000218 | 4.661543506 | -4.477847 | 2.369233 | down | -1.8855539 | leptin receptor overlapping transcript///leptin receptor |
| 384 | FTCD | 0.000815 | 0.0000953 | 4.020907099 | -4.082884 | 0.9763734 | down | -1.8931003 | formimidoyltransferase cyclodeaminase |
| 385 | ACADL | 0.00231 | 0.00038 | 3.420216403 | -3.691165 | -0.3167834 | down | -1.8964809 | acyl-CoA dehydrogenase, long chain |
| 386 | ABCA8 | 0.0184 | 0.00565 | 2.247951552 | -2.834345 | -2.7940535 | down | -1.8969901 | ATP binding cassette subfamily A member 8 |
| 387 | CNTN3 | 0.0121 | 0.0033 | 2.48148606 | -3.018117 | -2.3066532 | down | -1.9065523 | contactin 3 |
| 388 | CPNE6 | 4.8E-21 | 6.14E-25 | 24.21183163 | -14.254998 | 45.6404922 | down | -1.9102697 | copine 6 |
| 389 | SPATA18 | 0.000425 | 0.0000393 | 4.40560745 | -4.322693 | 1.8120161 | down | -1.9119351 | spermatogenesis associated 18 |
| 390 | EFHD1 | 0.000179 | 0.0000122 | 4.913640169 | -4.62754 | 2.9185337 | down | -1.9253528 | EF-hand domain family member D1 |
| 391 | AFM | 0.00413 | 0.000812 | 3.090443971 | -3.464128 | -1.0223973 | down | -1.9285363 | afamin |
| 392 | HBA2///HBA1 | 0.0000373 | 0.0000015 | 5.823908741 | -5.148339 | 4.911934 | down | -1.9293302 | hemoglobin subunit alpha 2///hemoglobin subunit alpha 1 |
| 393 | ASS1 | 2.79E-09 | 9.33E-12 | 11.03011836 | -7.810205 | 16.4561743 | down | -1.9302485 | argininosuccinate synthase 1 |
| 394 | GSPT2 | 0.00402 | 0.000785 | 3.105130343 | -3.474413 | -0.9911629 | down | -1.9330279 | G1 to S phase transition 2 |
| 395 | CCBE1 | 1.64E-14 | 1.23E-17 | 16.91009489 | -10.625947 | 29.5321273 | down | -1.9357832 | collagen and calcium binding EGF domains 1 |
| 396 | PSAT1 | 0.000166 | 0.000011 | 4.958607315 | -4.653452 | 3.0147506 | down | -1.9385126 | phosphoserine aminotransferase 1 |
| 397 | ST6GAL2 | 8.87E-16 | 5.19E-19 | 18.28483264 | -11.289745 | 32.579828 | down | -1.9387053 | ST6 beta-galactoside alpha-2,6-sialyltransferase 2 |
| 398 | SLC7A2 | 0.000121 | 0.00000718 | 5.143875556 | -4.761985 | 3.421255 | down | -1.9405285 | solute carrier family 7 member 2 |
| 399 | HHIP | 4.55E-09 | 1.62E-11 | 10.79048499 | -7.69446 | 15.9251224 | down | -1.9410144 | hedgehog interacting protein |
| 400 | PTPRS | 0.0000352 | 0.00000139 | 5.8569852 | -5.166927 | 4.9852711 | down | -1.9523734 | protein tyrosine phosphatase, receptor type S |
| 401 | SULT1E1 | 0.00235 | 0.000389 | 3.410050399 | -3.68381 | -0.3401625 | down | -1.9537436 | sulfotransferase family 1E member 1 |
| 402 | FOSB | 0.0000271 | 0.000000963 | 6.016373713 | -5.255679 | 5.3373864 | down | -1.9709017 | FosB proto-oncogene, AP-1 transcription factor subunit |
| 403 | TACSTD2 | 0.000569 | 0.0000581 | 4.235823868 | -4.217799 | 1.4425825 | down | -1.9777013 | tumor-associated calcium signal transducer 2 |
| 404 | PCK1 | 0.00573 | 0.00125 | 2.903089987 | -3.331884 | -1.4176437 | down | -1.9838722 | phosphoenolpyruvate carboxykinase 1 |
| 405 | COLEC11 | 0.000189 | 0.0000132 | 4.879426069 | -4.607873 | 2.8457309 | down | -1.9840435 | collectin subfamily member 11 |
| 406 | MCC | 0.00155 | 0.000224 | 3.649751982 | -3.843482 | 0.1750117 | down | -1.9841394 | mutated in colorectal cancers |
| 407 | MBL2 | 0.0109 | 0.00287 | 2.542118103 | -3.063635 | -2.1820625 | down | -1.9843912 | mannose binding lectin 2 |
| 408 | KLKB1 | 0.0000331 | 0.00000126 | 5.899629455 | -5.190071 | 5.076785 | down | -1.9849521 | kallikrein B1 |
| 409 | SHBG | 0.00000168 | 2.36E-08 | 7.627087997 | -6.114774 | 8.8932728 | down | -1.9865289 | sex hormone binding globulin |
| 410 | GADD45G | 0.00000596 | 0.000000131 | 6.882728704 | -5.724185 | 7.2457539 | down | -1.9872861 | growth arrest and DNA damage inducible gamma |
| 411 | C3P1 | 0.0058 | 0.00127 | 2.896196279 | -3.326402 | -1.4337709 | down | -1.9911073 | complement component 3 precursor pseudogene |
| 412 | ZG16 | 0.0000693 | 0.00000344 | 5.463441557 | -4.946035 | 4.1231579 | down | -2.0000054 | zymogen granule protein 16 |
| 413 | IGFBP3 | 0.00001 | 0.000000256 | 6.591760035 | -5.568795 | 6.604009 | down | -2.0033122 | insulin like growth factor binding protein 3 |
| 414 | ADH1B | 0.0153 | 0.00447 | 2.349692477 | -2.915113 | -2.5829533 | down | -2.0185356 | alcohol dehydrogenase 1B (class I), beta polypeptide |
| 415 | SLC1A2 | 0.0186 | 0.00573 | 2.241845378 | -2.829707 | -2.8060242 | down | -2.0203307 | solute carrier family 1 member 2 |
| 416 | CPEB3 | 0.00000137 | 1.84E-08 | 7.735182177 | -6.171655 | 9.1369789 | down | -2.0216224 | cytoplasmic polyadenylation element binding protein 3 |
| 417 | CFHR3 | 0.00827 | 0.002 | 2.698970004 | -3.181483 | -1.8525168 | down | -2.0253103 | complement factor H related 3 |
| 418 | ANK3 | 0.00314 | 0.000568 | 3.245651664 | -3.572277 | -0.6904644 | down | -2.0322648 | ankyrin 3, node of Ranvier (ankyrin G) |
| 419 | LECT2 | 0.0206 | 0.00655 | 2.1837587 | -2.782965 | -2.9257641 | down | -2.0371249 | leukocyte cell derived chemotaxin 2 |
| 420 | CCL15-CCL14///CCL14 | 1.82E-08 | 8.24E-11 | 10.08407279 | -7.349245 | 14.3508132 | down | -2.0482306 | CCL15-CCL14 readthrough (NMD candidate)///C-C motif chemokine ligand 14 |
| 421 | PEG3 | 0.0133 | 0.00371 | 2.43062609 | -2.978444 | -2.4140046 | down | -2.0545352 | paternally expressed 3 |
| 422 | KCND3 | 0.000866 | 0.000103 | 3.987162775 | -4.060624 | 0.9004416 | down | -2.0630372 | potassium voltage-gated channel subfamily D member 3 |
| 423 | FETUB | 0.0162 | 0.00479 | 2.319664487 | -2.891826 | -2.6443234 | down | -2.0644688 | fetuin B |
| 424 | PPP1R1A | 0.00175 | 0.000264 | 3.578396073 | -3.796192 | 0.0207847 | down | -2.0722062 | protein phosphatase 1 regulatory inhibitor subunit 1A |
| 425 | PAIP2B | 0.00000266 | 4.31E-08 | 7.36552273 | -5.979034 | 8.3154392 | down | -2.0766593 | poly(A) binding protein interacting protein 2B |
| 426 | HPGD | 0.0596 | 0.0254 | 1.595166283 | -2.272751 | -4.1206037 | down | -2.078629 | hydroxyprostaglandin dehydrogenase 15-(NAD) |
| 427 | SLC4A4 | 0.00696 | 0.00161 | 2.793174124 | -3.251969 | -1.6506835 | down | -2.0815536 | solute carrier family 4 member 4 |
| 428 | MAN1C1 | 0.0000826 | 0.00000433 | 5.363512104 | -4.888861 | 3.9034696 | down | -2.0849705 | mannosidase alpha class 1C member 1 |
| 429 | PRG4 | 0.00443 | 0.000892 | 3.049635146 | -3.435516 | -1.1089142 | down | -2.0860743 | proteoglycan 4 |
| 430 | RBP1 | 0.00294 | 0.000522 | 3.282329497 | -3.597257 | -0.6127021 | down | -2.09616 | retinol binding protein 1 |
| 431 | ADH1C | 0.0193 | 0.00603 | 2.219682688 | -2.811841 | -2.8519904 | down | -2.102754 | alcohol dehydrogenase 1C (class I), gamma polypeptide |
| 432 | PLG | 0.000304 | 0.0000252 | 4.598599459 | -4.43988 | 2.2317162 | down | -2.1107026 | plasminogen |
| 433 | HMGCS2 | 0.00000273 | 4.47E-08 | 7.349692477 | -5.97069 | 8.2800973 | down | -2.1119706 | 3-hydroxy-3-methylglutaryl-CoA synthase 2 |
| 434 | C7 | 0.0239 | 0.00793 | 2.100726813 | -2.714741 | -3.0975157 | down | -2.1150695 | complement component 7 |
| 435 | CYP4A11 | 0.0000204 | 0.00000067 | 6.173925197 | -5.342477 | 5.6847832 | down | -2.1168206 | cytochrome P450 family 4 subfamily A member 11 |
| 436 | LGSN | 0.000138 | 0.00000851 | 5.07007044 | -4.71919 | 3.2602983 | down | -2.1463233 | lengsin, lens protein with glutamine synthetase domain |
| 437 | CYP3A43 | 3.71E-08 | 1.95E-10 | 9.709965389 | -7.164939 | 13.5171549 | down | -2.1532166 | cytochrome P450 family 3 subfamily A member 43 |
| 438 | HBB | 0.00000669 | 0.000000151 | 6.821023053 | -5.692046 | 7.1123465 | down | -2.1565735 | hemoglobin subunit beta |
| 439 | WDR72 | 0.0199 | 0.00625 | 2.204119983 | -2.799437 | -2.8837592 | down | -2.1605243 | WD repeat domain 72 |
| 440 | LINC01018 | 0.00226 | 0.00037 | 3.431798276 | -3.698929 | -0.2920675 | down | -2.1613777 | long intergenic non-protein coding RNA 1018 |
| 441 | LY6E | 0.0000179 | 0.000000558 | 6.253365801 | -5.385774 | 5.8591611 | down | -2.1625519 | lymphocyte antigen 6 complex, locus E |
| 442 | ANGPTL1 | 3.28E-08 | 1.71E-10 | 9.76700389 | -7.193454 | 13.6457864 | down | -2.1637562 | angiopoietin like 1 |
| 443 | KMO | 0.000498 | 0.0000487 | 4.312471039 | -4.265202 | 1.608793 | down | -2.1736746 | kynurenine 3-monooxygenase (kynurenine 3-hydroxylase) |
| 444 | HOGA1 | 0.00016 | 0.0000105 | 4.978810701 | -4.665654 | 3.0601706 | down | -2.1761898 | 4-hydroxy-2-oxoglutarate aldolase 1 |
| 445 | FLJ22763 | 0.0000959 | 0.00000531 | 5.274905479 | -4.837675 | 3.708039 | down | -2.1769853 | uncharacterized LOC401081 |
| 446 | CPED1 | 0.000255 | 0.0000198 | 4.70333481 | -4.502717 | 2.4597133 | down | -2.1781015 | cadherin like and PC-esterase domain containing 1 |
| 447 | STAB2 | 1.55E-16 | 8.2E-20 | 19.08618615 | -11.680126 | 34.3550061 | down | -2.179413 | stabilin 2 |
| 448 | NPY1R | 2.88E-09 | 9.81E-12 | 11.00833099 | -7.799529 | 16.4071326 | down | -2.186392 | neuropeptide Y receptor Y1 |
| 449 | TUBE1 | 1.77E-08 | 7.85E-11 | 10.10513034 | -7.359495 | 14.3973267 | down | -2.1919219 | tubulin epsilon 1 |
| 450 | CD5L | 2.84E-09 | 9.6E-12 | 11.01772877 | -7.804148 | 16.4283503 | down | -2.1928942 | CD5 molecule like |
| 451 | CTH | 0.000571 | 0.0000585 | 4.232844134 | -4.215805 | 1.4356163 | down | -2.1929184 | cystathionine gamma-lyase |
| 452 | FAM13A | 0.0000073 | 0.00000017 | 6.769551079 | -5.664432 | 6.9979998 | down | -2.1929871 | family with sequence similarity 13 member A |
| 453 | MT2A | 0.000189 | 0.0000132 | 4.879426069 | -4.608011 | 2.8462397 | down | -2.1950784 | metallothionein 2A |
| 454 | FOS | 0.00059 | 0.0000611 | 4.21395879 | -4.204251 | 1.3953052 | down | -2.2021729 | Fos proto-oncogene, AP-1 transcription factor subunit |
| 455 | UGT2B17 | 0.0479 | 0.0192 | 1.716698771 | -2.382994 | -3.880282 | down | -2.2065156 | UDP glucuronosyltransferase family 2 member B17 |
| 456 | PDGFRA | 0.00647 | 0.00146 | 2.835647144 | -3.282085 | -1.5633832 | down | -2.2094204 | platelet derived growth factor receptor alpha |
| 457 | SPP2 | 0.00122 | 0.000163 | 3.787812396 | -3.933284 | 0.4716277 | down | -2.2119137 | secreted phosphoprotein 2 |
| 458 | HGFAC | 0.00416 | 0.000819 | 3.086716098 | -3.461735 | -1.0296519 | down | -2.2192764 | HGF activator |
| 459 | HSD11B1 | 0.0169 | 0.00507 | 2.294992041 | -2.871996 | -2.6962606 | down | -2.2279527 | hydroxysteroid 11-beta dehydrogenase 1 |
| 460 | CYP1A1 | 0.00525 | 0.00111 | 2.954677021 | -3.367783 | -1.3115259 | down | -2.2308973 | cytochrome P450 family 1 subfamily A member 1 |
| 461 | UGT3A1 | 0.0146 | 0.00417 | 2.379863945 | -2.938667 | -2.5204666 | down | -2.2332576 | UDP glycosyltransferase family 3 member A1 |
| 462 | KAZN | 7.04E-10 | 1.69E-12 | 11.7721133 | -8.168818 | 18.1097164 | down | -2.2335377 | kazrin, periplakin interacting protein |
| 463 | KDM8 | 3.04E-10 | 6E-13 | 12.22184875 | -8.384259 | 19.1078829 | down | -2.2381675 | lysine demethylase 8 |
| 464 | SLC10A1 | 0.00649 | 0.00147 | 2.832682665 | -3.280141 | -1.5690366 | down | -2.2564259 | solute carrier family 10 member 1 |
| 465 | FAM65C | 4.45E-14 | 3.74E-17 | 16.4271284 | -10.393933 | 28.4596458 | down | -2.2608238 | family with sequence similarity 65 member C |
| 466 | TDO2 | 0.0064 | 0.00144 | 2.841637508 | -3.286503 | -1.5505233 | down | -2.268624 | tryptophan 2,3-dioxygenase |
| 467 | MASP1 | 1.05E-09 | 2.76E-12 | 11.55909092 | -8.065354 | 17.6315147 | down | -2.2733166 | mannan binding lectin serine peptidase 1 |
| 468 | FITM1 | 4.7E-14 | 4.04E-17 | 16.39361863 | -10.377978 | 28.3857768 | down | -2.2815543 | fat storage inducing transmembrane protein 1 |
| 469 | PHGDH | 0.00000161 | 2.23E-08 | 7.651695137 | -6.127782 | 8.9489248 | down | -2.2839022 | phosphoglycerate dehydrogenase |
| 470 | ALDOB | 0.00113 | 0.000146 | 3.835647144 | -3.96481 | 0.5769004 | down | -2.3107154 | aldolase, fructose-bisphosphate B |
| 471 | CETP | 1.51E-09 | 4.21E-12 | 11.3757179 | -7.977349 | 17.2254527 | down | -2.3141964 | cholesteryl ester transfer protein |
| 472 | LOC101928916///NNMT | 0.0113 | 0.003 | 2.522878745 | -3.049047 | -2.2221577 | down | -2.3159899 | uncharacterized LOC101928916///nicotinamide N-methyltransferase |
| 473 | STEAP4 | 0.0000328 | 0.00000125 | 5.903089987 | -5.192814 | 5.0876459 | down | -2.3168839 | STEAP4 metalloreductase |
| 474 | SRD5A2 | 0.0000249 | 0.000000862 | 6.064492734 | -5.282242 | 5.4433884 | down | -2.3183348 | steroid 5 alpha-reductase 2 |
| 475 | MT1HL1 | 0.0000602 | 0.00000286 | 5.543633967 | -4.991362 | 4.2983534 | down | -2.3227456 | metallothionein 1H-like 1 |
| 476 | MFAP3L | 0.00346 | 0.000646 | 3.189767482 | -3.533358 | -0.8108012 | down | -2.3357336 | microfibrillar associated protein 3 like |
| 477 | LOC100287413///GLYATL1 | 0.0012 | 0.000158 | 3.801342913 | -3.941569 | 0.499234 | down | -2.3404287 | uncharacterized LOC100287413///glycine-N-acyltransferase like 1 |
| 478 | ANXA10 | 0.000332 | 0.0000281 | 4.55129368 | -4.41072 | 2.1266084 | down | -2.3478072 | annexin A10 |
| 479 | FOLH1B///FOLH1 | 0.0000244 | 0.000000841 | 6.075204004 | -5.288135 | 5.4669404 | down | -2.386906 | folate hydrolase 1B///folate hydrolase (prostate-specific membrane antigen) 1 |
| 480 | SLC16A10 | 0.000000878 | 1.01E-08 | 7.995678626 | -6.304393 | 9.7091204 | down | -2.3925866 | solute carrier family 16 member 10 |
| 481 | GSTZ1 | 2.73E-08 | 1.38E-10 | 9.860120914 | -7.239358 | 13.8531333 | down | -2.4068441 | glutathione S-transferase zeta 1 |
| 482 | OLFML3 | 0.00000361 | 6.65E-08 | 7.177178355 | -5.880351 | 7.8988042 | down | -2.4118445 | olfactomedin like 3 |
| 483 | BHMT | 0.00232 | 0.000383 | 3.416801226 | -3.688761 | -0.3244308 | down | -2.4227383 | betaine--homocysteine S-methyltransferase |
| 484 | CYP2C9 | 0.00721 | 0.00168 | 2.774690718 | -3.237702 | -1.6918192 | down | -2.4253025 | cytochrome P450 family 2 subfamily C member 9 |
| 485 | KBTBD11 | 0.000000047 | 2.67E-10 | 9.573488739 | -7.097877 | 13.2151695 | down | -2.4275091 | kelch repeat and BTB domain containing 11 |
| 486 | IGF1 | 0.00229 | 0.000375 | 3.425968732 | -3.694893 | -0.3049213 | down | -2.4339249 | insulin like growth factor 1 |
| 487 | MARCO | 5.14E-13 | 5.08E-16 | 15.29413629 | -9.852093 | 25.9442163 | down | -2.4340483 | macrophage receptor with collagenous structure |
| 488 | CXCL2 | 0.000716 | 0.00008 | 4.096910013 | -4.130977 | 1.141385 | down | -2.4428222 | C-X-C motif chemokine ligand 2 |
| 489 | FBP1 | 0.00000887 | 0.00000022 | 6.657577319 | -5.6045 | 6.7507281 | down | -2.4435593 | fructose-bisphosphatase 1 |
| 490 | GPR88 | 0.0283 | 0.00989 | 2.004803708 | -2.634966 | -3.2937521 | down | -2.458216 | G protein-coupled receptor 88 |
| 491 | SOCS2 | 0.00000237 | 3.69E-08 | 7.432973634 | -6.014394 | 8.4654454 | down | -2.4661802 | suppressor of cytokine signaling 2 |
| 492 | S100A8 | 0.0000143 | 0.000000414 | 6.382999659 | -5.456237 | 6.1444684 | down | -2.479087 | S100 calcium binding protein A8 |
| 493 | FOLH1B | 0.00000201 | 0.00000003 | 7.522878745 | -6.061508 | 8.6658872 | down | -2.4792544 | folate hydrolase 1B |
| 494 | MOGAT2 | 0.00000449 | 8.98E-08 | 7.046723663 | -5.811601 | 7.6103384 | down | -2.4870201 | monoacylglycerol O-acyltransferase 2 |
| 495 | CTNNA3 | 3.48E-11 | 4.96E-14 | 13.30451832 | -8.902451 | 21.5181849 | down | -2.4892152 | catenin alpha 3 |
| 496 | EXPH5 | 0.0000834 | 0.00000438 | 5.358525889 | -4.885605 | 3.8910014 | down | -2.5131853 | exophilin 5 |
| 497 | RDH16 | 0.0000438 | 0.00000188 | 5.725842151 | -5.094564 | 4.700567 | down | -2.5272165 | retinol dehydrogenase 16 (all-trans) |
| 498 | LINC01554 | 0.000667 | 0.000072 | 4.142667504 | -4.159524 | 1.2399511 | down | -2.5376564 | long intergenic non-protein coding RNA 1554 |
| 499 | CDH19 | 1.01E-08 | 4.06E-11 | 10.39147397 | -7.499384 | 15.0336007 | down | -2.5399616 | cadherin 19 |
| 500 | CYP4A22///CYP4A11 | 0.0000716 | 0.00000358 | 5.446116973 | -4.935874 | 4.0840068 | down | -2.548151 | cytochrome P450 family 4 subfamily A member 22///cytochrome P450 family 4 subfamily A member 11 |
| 501 | CYP2C8 | 0.00167 | 0.000247 | 3.607303047 | -3.815678 | 0.0841674 | down | -2.5486294 | cytochrome P450 family 2 subfamily C member 8 |
| 502 | APOA5 | 6.87E-09 | 2.61E-11 | 10.58335949 | -7.592993 | 15.4608256 | down | -2.5505728 | apolipoprotein A5 |
| 503 | HGF | 0.000053 | 0.00000242 | 5.616184634 | -5.032664 | 4.458776 | down | -2.5675364 | hepatocyte growth factor |
| 504 | RSPO3 | 5.12E-17 | 2.25E-20 | 19.64781748 | -11.955531 | 35.5985677 | down | -2.5748069 | R-spondin 3 |
| 505 | LIFR | 9.22E-08 | 5.79E-10 | 9.237321436 | -6.931442 | 12.4690692 | down | -2.5907697 | leukemia inhibitory factor receptor alpha |
| 506 | FXYD1 | 0.0000848 | 0.00000448 | 5.348721986 | -4.880159 | 3.8701608 | down | -2.6060439 | FXYD domain containing ion transport regulator 1 |
| 507 | IDO2 | 6.84E-09 | 2.58E-11 | 10.58838029 | -7.595768 | 15.4735079 | down | -2.612797 | indoleamine 2,3-dioxygenase 2 |
| 508 | PLAC8 | 5.62E-10 | 1.29E-12 | 11.88941029 | -8.224805 | 18.3688171 | down | -2.616075 | placenta specific 8 |
| 509 | AADAT | 4.5E-10 | 9.96E-13 | 12.00174066 | -8.278717 | 18.6185135 | down | -2.6218639 | aminoadipate aminotransferase |
| 510 | TENM1 | 0.000193 | 0.0000135 | 4.869666232 | -4.600872 | 2.8198593 | down | -2.6327921 | teneurin transmembrane protein 1 |
| 511 | MME | 0.00513 | 0.00108 | 2.966576245 | -3.376827 | -1.2846517 | down | -2.6333213 | membrane metallo-endopeptidase |
| 512 | DOK5 | 1.55E-10 | 2.75E-13 | 12.56066731 | -8.546419 | 19.860976 | down | -2.6537017 | docking protein 5 |
| 513 | ECM1 | 4.09E-19 | 1.2E-22 | 21.92081875 | -13.08534 | 40.6136018 | down | -2.6541969 | extracellular matrix protein 1 |
| 514 | SRPX | 0.0000634 | 0.00000304 | 5.517126416 | -4.975913 | 4.2385409 | down | -2.6642739 | sushi repeat containing protein, X-linked |
| 515 | GYS2 | 0.0037 | 0.000704 | 3.152427341 | -3.507475 | -0.890281 | down | -2.6656324 | glycogen synthase 2 |
| 516 | ZGPAT///LIME1 | 3.72E-09 | 1.29E-11 | 10.88941029 | -7.741545 | 16.1409798 | down | -2.6669444 | zinc finger CCCH-type and G-patch domain containing///Lck interacting transmembrane adaptor 1 |
| 517 | ADH4 | 0.0127 | 0.0035 | 2.455931956 | -2.998321 | -2.3603633 | down | -2.6819619 | alcohol dehydrogenase 4 (class II), pi polypeptide |
| 518 | MT1X | 0.0000748 | 0.00000381 | 5.419075024 | -4.92032 | 4.024169 | down | -2.6874147 | metallothionein 1X |
| 519 | CYP2B7P | 0.000134 | 0.00000823 | 5.084600165 | -4.727569 | 3.2917452 | down | -2.6938965 | cytochrome P450 family 2 subfamily B member 7, pseudogene |
| 520 | ZGPAT | 1.74E-09 | 5.32E-12 | 11.27408837 | -7.928 | 16.9980491 | down | -2.6949912 | zinc finger CCCH-type and G-patch domain containing |
| 521 | CYP2A7 | 0.000288 | 0.0000234 | 4.630784143 | -4.458689 | 2.2997517 | down | -2.6979831 | cytochrome P450 family 2 subfamily A member 7 |
| 522 | TFPI2 | 9.51E-08 | 6.04E-10 | 9.218963061 | -6.922031 | 12.427036 | down | -2.7035488 | tissue factor pathway inhibitor 2 |
| 523 | CYP8B1 | 0.0054 | 0.00116 | 2.935542011 | -3.35537 | -1.348319 | down | -2.7049548 | cytochrome P450 family 8 subfamily B member 1 |
| 524 | AKR1D1 | 0.00142 | 0.0002 | 3.698970004 | -3.875755 | 0.2810503 | down | -2.7163565 | aldo-keto reductase family 1, member D1 |
| 525 | GLYAT | 0.00015 | 0.00000964 | 5.015922966 | -4.687479 | 3.1415906 | down | -2.723765 | glycine-N-acyltransferase |
| 526 | GPM6A | 7.23E-11 | 1.18E-13 | 12.92811799 | -8.723154 | 20.6830968 | down | -2.7268606 | glycoprotein M6A |
| 527 | LYVE1 | 1.95E-14 | 1.54E-17 | 16.81247928 | -10.57964 | 29.3183378 | down | -2.7300706 | lymphatic vessel endothelial hyaluronan receptor 1 |
| 528 | ACSM3 | 0.00000166 | 2.33E-08 | 7.632644079 | -6.118165 | 8.9077774 | down | -2.7332518 | acyl-CoA synthetase medium-chain family member 3 |
| 529 | IL13RA2 | 1.53E-09 | 4.32E-12 | 11.36451625 | -7.971819 | 17.1999561 | down | -2.7385868 | interleukin 13 receptor subunit alpha 2 |
| 530 | CAPN3 | 2.7E-13 | 2.57E-16 | 15.59006688 | -9.993354 | 26.6012049 | down | -2.7432541 | calpain 3 |
| 531 | MFSD2A | 0.0000112 | 0.000000298 | 6.525783736 | -5.533908 | 6.4610882 | down | -2.7438075 | major facilitator superfamily domain containing 2A |
| 532 | MT1H | 0.0000428 | 0.00000181 | 5.742321425 | -5.103259 | 4.7346597 | down | -2.7529825 | metallothionein 1H |
| 533 | ASPA | 2.79E-09 | 9.34E-12 | 11.02965312 | -7.809911 | 16.4548254 | down | -2.755413 | aspartoacylase |
| 534 | GHR | 0.0000198 | 0.000000643 | 6.191789027 | -5.352117 | 5.7235441 | down | -2.7733676 | growth hormone receptor |
| 535 | IGFALS | 2.8E-14 | 2.31E-17 | 16.63638802 | -10.494881 | 28.9266705 | down | -2.7819862 | insulin like growth factor binding protein acid labile subunit |
| 536 | SLC16A4 | 0.000000183 | 1.37E-09 | 8.863279433 | -6.744975 | 11.6393857 | down | -2.7962823 | solute carrier family 16 member 4 |
| 537 | MAGI2-AS3 | 0.000000154 | 1.12E-09 | 8.950781977 | -6.787846 | 11.8295234 | down | -2.8053292 | MAGI2 antisense RNA 3 |
| 538 | INS-IGF2///IGF2 | 0.0208 | 0.00665 | 2.177178355 | -2.777464 | -2.9397462 | down | -2.8224679 | INS-IGF2 readthrough///insulin like growth factor 2 |
| 539 | CYP2A6 | 0.00258 | 0.00044 | 3.356547324 | -3.647765 | -0.4542467 | down | -2.8367153 | cytochrome P450 family 2 subfamily A member 6 |
| 540 | FREM2 | 2.93E-13 | 2.84E-16 | 15.54668166 | -9.972697 | 26.5051757 | down | -2.8381097 | FRAS1 related extracellular matrix protein 2 |
| 541 | DCN | 0.0017 | 0.000254 | 3.595166283 | -3.807602 | 0.0578699 | down | -2.8472417 | decorin |
| 542 | VNN1 | 0.00281 | 0.000494 | 3.306273051 | -3.613608 | -0.5615862 | down | -2.8653018 | vanin 1 |
| 543 | SLC25A47 | 0.000013 | 0.000000365 | 6.437707136 | -5.485831 | 6.2648474 | down | -2.8878652 | solute carrier family 25 member 47 |
| 544 | LCAT | 0.000000349 | 3.16E-09 | 8.500312917 | -6.562053 | 10.8325065 | down | -2.9090524 | lecithin-cholesterol acyltransferase |
| 545 | BBOX1 | 0.000237 | 0.0000178 | 4.749579998 | -4.529948 | 2.5591412 | down | -2.9118883 | gamma-butyrobetaine hydroxylase 1 |
| 546 | ADGRG7 | 0.00000071 | 7.75E-09 | 8.110698297 | -6.363805 | 9.9666998 | down | -2.9489073 | adhesion G protein-coupled receptor G7 |
| 547 | SLCO4C1 | 0.000000356 | 3.24E-09 | 8.48945499 | -6.556359 | 10.8075098 | down | -2.9620184 | solute carrier organic anion transporter family member 4C1 |
| 548 | BCHE | 0.00289 | 0.000511 | 3.2915791 | -3.603377 | -0.5935925 | down | -2.9868039 | butyrylcholinesterase |
| 549 | CYP2B7P///CYP2B6 | 0.000167 | 0.0000112 | 4.950781977 | -4.649647 | 3.0006005 | down | -3.0173919 | cytochrome P450 family 2 subfamily B member 7, pseudogene///cytochrome P450 family 2 subfamily B member 6 |
| 550 | LPA | 0.000000448 | 4.28E-09 | 8.368556231 | -6.495068 | 10.5389097 | down | -3.0178191 | lipoprotein(a) |
| 551 | DIRAS3 | 1.74E-09 | 5.34E-12 | 11.27245874 | -7.927186 | 16.9942998 | down | -3.0213855 | DIRAS family GTPase 3 |
| 552 | GNMT | 0.000129 | 0.00000777 | 5.109578981 | -4.742142 | 3.3465148 | down | -3.0278921 | glycine N-methyltransferase |
| 553 | MT1F | 0.00000589 | 0.000000129 | 6.88941029 | -5.727633 | 7.2600846 | down | -3.0308432 | metallothionein 1F |
| 554 | CXCL12 | 0.0000654 | 0.00000319 | 5.496209317 | -4.964762 | 4.19543 | down | -3.0427731 | C-X-C motif chemokine ligand 12 |
| 555 | IL1RAP | 8.46E-09 | 3.33E-11 | 10.47755577 | -7.541905 | 15.2275271 | down | -3.0490054 | interleukin 1 receptor accessory protein |
| 556 | BCO2 | 9.46E-08 | 5.99E-10 | 9.222573178 | -6.924081 | 12.4361907 | down | -3.107234 | beta-carotene oxygenase 2 |
| 557 | DNASE1L3 | 2.26E-08 | 1.1E-10 | 9.958607315 | -7.288376 | 14.0749102 | down | -3.1100238 | deoxyribonuclease 1 like 3 |
| 558 | NAT2 | 0.000000889 | 1.03E-08 | 7.987162775 | -6.300139 | 9.6907119 | down | -3.1256514 | N-acetyltransferase 2 |
| 559 | CLRN3 | 0.000518 | 0.0000514 | 4.289036881 | -4.250553 | 1.5572969 | down | -3.1431812 | clarin 3 |
| 560 | APOF | 0.00013 | 0.00000786 | 5.104577454 | -4.739121 | 3.3351546 | down | -3.1457355 | apolipoprotein F |
| 561 | MT1G | 0.0000094 | 0.000000236 | 6.627087997 | -5.588131 | 6.6834076 | down | -3.1507748 | metallothionein 1G |
| 562 | GLS2 | 0.000114 | 0.00000666 | 5.176525771 | -4.780911 | 3.4927156 | down | -3.1880113 | glutaminase 2 |
| 563 | CYP39A1 | 0.000429 | 0.0000397 | 4.401209493 | -4.31934 | 1.8001171 | down | -3.1894537 | cytochrome P450 family 39 subfamily A member 1 |
| 564 | CYP3A4 | 0.000413 | 0.0000379 | 4.42136079 | -4.331908 | 1.8447553 | down | -3.2242164 | cytochrome P450 family 3 subfamily A member 4 |
| 565 | CYP2B6 | 0.0000154 | 0.000000455 | 6.341988603 | -5.434139 | 6.0547943 | down | -3.2455216 | cytochrome P450 family 2 subfamily B member 6 |
| 566 | ESR1 | 0.000000966 | 1.16E-08 | 7.935542011 | -6.273605 | 9.5759965 | down | -3.2832618 | estrogen receptor 1 |
| 567 | FAM134B | 0.00000673 | 0.000000152 | 6.818156412 | -5.690275 | 7.1050019 | down | -3.3288953 | family with sequence similarity 134 member B |
| 568 | CLEC4M | 2.89E-24 | 1.26E-28 | 27.89962945 | -16.222405 | 53.6767726 | down | -3.3860434 | C-type lectin domain family 4 member M |
| 569 | GBA3 | 0.000224 | 0.0000166 | 4.779891912 | -4.548262 | 2.6262248 | down | -3.4439108 | glucosylceramidase beta 3 (gene/pseudogene) |
| 570 | UPP2 | 0.00000479 | 9.78E-08 | 7.009661145 | -5.792057 | 7.5286078 | down | -3.4835998 | uridine phosphorylase 2 |
| 571 | THRSP | 0.0018 | 0.000274 | 3.562249437 | -3.785912 | -0.0125625 | down | -3.4936956 | thyroid hormone responsive |
| 572 | TTC36 | 0.000000127 | 8.75E-10 | 9.057991947 | -6.841944 | 12.0699894 | down | -3.5318765 | tetratricopeptide repeat domain 36 |
| 573 | C9 | 0.00894 | 0.00222 | 2.653647026 | -3.148888 | -1.9446623 | down | -3.6295775 | complement component 9 |
| 574 | TMEM27 | 0.00000023 | 1.85E-09 | 8.732828272 | -6.678478 | 11.3452202 | down | -3.657474 | transmembrane protein 27 |
| 575 | AVPR1A | 0.0000937 | 0.00000515 | 5.288192771 | -4.845604 | 3.7382344 | down | -3.6822251 | arginine vasopressin receptor 1A |
| 576 | HAO2 | 0.00000458 | 9.21E-08 | 7.03574037 | -5.805895 | 7.5864657 | down | -3.7105575 | hydroxyacid oxidase 2 |
| 577 | OAT | 0.000000843 | 9.63E-09 | 8.016373713 | -6.315565 | 9.7574867 | down | -3.7537137 | ornithine aminotransferase |
| 578 | SLC22A1 | 0.000115 | 0.00000671 | 5.17327748 | -4.779235 | 3.4863804 | down | -3.8262054 | solute carrier family 22 member 1 |
| 579 | CYP26A1 | 1.58E-11 | 2.06E-14 | 13.68613278 | -9.084918 | 22.3687253 | down | -3.8452016 | cytochrome P450 family 26 subfamily A member 1 |
| 580 | LINC00844 | 0.000293 | 0.000024 | 4.619788758 | -4.452406 | 2.2770048 | down | -3.878432 | long intergenic non-protein coding RNA 844 |
| 581 | CXCL14 | 5.12E-17 | 2.16E-20 | 19.66554625 | -11.963901 | 35.6362411 | down | -3.9522003 | C-X-C motif chemokine ligand 14 |
| 582 | CYP1A2 | 0.00000873 | 0.000000215 | 6.66756154 | -5.610106 | 6.7738034 | down | -3.9869394 | cytochrome P450 family 1 subfamily A member 2 |
| 583 | CRHBP | 6.27E-12 | 7.23E-15 | 14.1408617 | -9.301725 | 23.3797252 | down | -4.0916639 | corticotropin releasing hormone binding protein |
| 584 | CLEC1B | 8.55E-19 | 2.66E-22 | 21.57511836 | -12.911233 | 39.8504656 | down | -4.1443201 | C-type lectin domain family 1 member B |
| 585 | PZP | 5.12E-17 | 2.15E-20 | 19.66756154 | -11.965257 | 35.642346 | down | -4.2017526 | PZP, alpha-2-macroglobulin like |
| 586 | FCN3 | 2.54E-12 | 2.83E-15 | 14.54821356 | -9.495646 | 24.2838988 | down | -4.2026258 | ficolin 3 |
| 587 | OIT3 | 1.35E-14 | 9.41E-18 | 17.02641038 | -10.682066 | 29.7910293 | down | -4.2163646 | oncoprotein induced transcript 3 |
| 588 | XIST | 0.000000647 | 6.84E-09 | 8.164943898 | -6.391508 | 10.087113 | down | -4.2288754 | X inactive specific transcript (non-protein coding) |
| 589 | LINC01093 | 1.53E-09 | 4.4E-12 | 11.35654732 | -7.967934 | 17.1820513 | down | -4.3472924 | long intergenic non-protein coding RNA 1093 |
| 590 | CLEC4G | 2.11E-22 | 2.32E-26 | 25.63451202 | -15.001489 | 48.7531882 | down | -4.350339 | C-type lectin domain family 4 member G |
| 591 | HAMP | 0.0000148 | 0.00000043 | 6.366531544 | -5.447403 | 6.1085994 | down | -4.4399953 | hepcidin antimicrobial peptide |
| 592 | CYP2C19 | 2.89E-24 | 1.59E-28 | 27.79860288 | -16.168113 | 53.4622975 | down | -4.5093412 | cytochrome P450 family 2 subfamily C member 19 |
| 593 | FCN2 | 1.08E-16 | 5.31E-20 | 19.27490548 | -11.772332 | 34.772199 | down | -4.5808137 | ficolin 2 |
| 594 | KCNN2 | 2.47E-20 | 3.61E-24 | 23.4424928 | -13.857867 | 43.9537458 | down | -4.7232954 | potassium calcium-activated channel subfamily N member 2 |
| 595 | SLCO1B3 | 0.0000122 | 0.000000335 | 6.474955193 | -5.506559 | 6.3493523 | down | -4.8250612 | solute carrier organic anion transporter family member 1B3 |
| 596 | ASCL1 | 0.000000119 | 8.01E-10 | 9.096367484 | -6.861205 | 12.1557449 | down | -4.9959728 | achaete-scute family bHLH transcription factor 1 |
| 597 | MT1M | 0.000000343 | 3.09E-09 | 8.510041521 | -6.56685 | 10.8535695 | down | -5.0437227 | metallothionein 1M |
| 598 | CNDP1 | 3.92E-10 | 8.31E-13 | 12.08039898 | -8.316489 | 18.7935738 | down | -5.1967917 | carnosine dipeptidase 1 |

**Supplementary table 2**
